# Spatiotemporal dynamics of odor responses in the lateral and dorsal olfactory bulb

**DOI:** 10.1371/journal.pbio.3000409

**Published:** 2019-09-18

**Authors:** Keeley L. Baker, Ganesh Vasan, Ankita Gumaste, Vincent A. Pieribone, Justus V. Verhagen

**Affiliations:** 1 The John B. Pierce Laboratory, New Haven, Connecticut, United States of America; 2 Department of Neuroscience, Yale School of Medicine, New Haven, Connecticut, United States of America; 3 Department of Cellular and Molecular Physiology, Yale School of Medicine, New Haven, Connecticut, United States of America; RWTH Aachen University, GERMANY

## Abstract

The mammalian olfactory bulb (OB) plays an essential role in odor processing during the perception of smell. Optical imaging of the OB has proven to be a key tool in elucidating the spatial odor mapping and temporal dynamics that underlie higher-order odor processing. Much is known about the activation of olfactory sensory neuron (OSN) glomerular responses in the dorsal olfactory bulb (dOB) during odor presentation. However, the dorsal bulb provides access to only approximately 25% of all glomeruli, and little is known about how the lateral bulb functions during this critical process. Here, we report, for the first time, simultaneous measurements of OSN glomerular activity from both the dOB and the lateral olfactory bulb (lOB), thus describing odor-specific spatial mapping and the temporal dynamics of olfactory input to both the dorsal and lateral bulb. Odor responses in the lateral bulb tended to be most prominent in the dorso-lateral (D-L) region. Lateral glomeruli became active in a dorso-ventral (D-V) sequence upon odor inhalation, unlike the anterio-posterior (A-P) activity wave typical of the dorsal glomeruli. Across the entire D-L bulb, the spatial organization of these dynamics can be explained neither by the purely mechanosensitive dynamics (to breathing clean air) nor by the response amplitudes across glomeruli. Instead, these dynamics can be explained by a combination of zonal receptor distributions, associated OB projections, and air flow paths across the epithelium upon inhalation. Remarkably, we also found that a subset of OSN glomeruli in the lOB was highly sensitive to extranasal air pressure changes, a response type that has not been reported in dorsal glomeruli.

## Abstract 2

The mammalian olfactory bulb plays an essential role in odor processing during the perception of smell, but most studies have focused on the dorsal olfactory bulb, which contains only a quarter of all glomeruli. In this study, imaging of the lateral olfactory bulb reveals new properties in smell processing.

## Introduction

Odor processing is critical for finding food and mates and detecting predators and is therefore vital for survival. Consequently, it is not surprising that, in mice, up to 5% of the protein coding genome is dedicated to the approximately 1,000 different olfactory receptors (ORs) present within the nasal epithelium, expressed by olfactory sensory neurons (OSNs) [1]. Each OSN expresses a single OR, and neurons expressing the same receptor are confined to one of 4 zones within the nose, albeit randomly located within these zones [2]. In the olfactory bulb (OB), the OSNs with a given receptor type map onto 1 or 2 glomeruli, one on the medial and one on the lateral surface, creating a mirror-symmetric glomerular map that wraps around the OB [3–6]. ORs can recognize multiple odorants, and the molecular features of an odorant can activate multiple ORs [7]. Molecular features of odorants also preferentially activate different olfactory epithelial zones [8, 9]. Spatial maps of glomerular activation have highlighted the topography of chemical properties of odorants in the OB [10]. The activity patterns of glomeruli are altered by the functional group of an odorant and its polarity, molecular shape (cyclic or noncyclic compounds), carbon chain length, concentration, and ortho- or retronasal route of entry of these odors [11–15].

Many of these functional mapping measurements have been performed using optical imaging from the dorsal olfactory bulb (dOB), where only approximately 25% of glomeruli can be accessed [12, 16–19]. The other parts of the bulb are not readily accessible, and thus there are limited data from nondorsal glomeruli. Dorsal glomeruli receive input only from the dorsal recess (zone 1) within the nasal cavity. Optical imaging of the lateral olfactory bulb (lOB) reports glomerular activation data from zones (2–4) of the epithelium [15]. All of the measurements from nondorsal glomeruli, to date, have been performed using population and activity–non-specific and low-speed imaging techniques, including intrinsic optical imaging [20], whole OB 2-deoxyglucose (2-DG) [21, 22], and functional magnetic resonance imaging (fMRI) [11]. These methods demonstrate time-averaged activity responses to odors. For example, activity patterns using 2-DG are obtained after a 45-minute exposure to a single odorant per animal, as the spatial maps are obtained in ex vivo brain slices. The spatial representations of hydrocarbons have been examined in the lateral bulb in rats by removing the eye and utilizing intrinsic optical imaging, highlighting two key areas of activation within the lateral bulb [20]. Intrinsic imaging does not relay direct information on neural firing rate, and therefore this study did not report response dynamics. This study also did not image the dorsal bulb simultaneously with the lateral bulb. Intrinsic imaging itself lacks neuronal specificity, as it monitors the hemodynamic response due not only to OSN input but also, for example, mitral cell/tufted cell output [23]. Although these techniques have given great insight into the spatial processing of odors [24], they are limited by their temporal resolution.

Although spatial bulbar odor maps are one aspect underlying odor perception, it is well established that the glomerular activation dynamics are also able to contribute to perception. Widefield calcium imaging affords high temporal resolution and the temporal glomerular dynamics of the dOB have been extensively described over the first respiratory cycle [25, 26], an important time window because a single sniff can be used for odor discrimination [27, 28]. The dynamic glomerular activation patterns unfolds over approximately 200 ms across the dorsal glomerular layer following inhalation during odor presentation [19]. Indeed, mice can discriminate glomerular input activity duration differences down to only 10 ms [29] and detect temporal optogenetic odor information down to 10 ms relative to the sniff cycle [30]. Furthermore, our lab has shown that mice are able to discriminate the temporal differences in optogenetic activation of spatially separated glomeruli across the dorsal bulb of only 13 ms, independently of sniff timing [31]. Mice were also shown to be able to discriminate optogenetic activation (“play back”) of dynamic imaged odor maps from the same maps rendered static, but with equal integrated optical power [31]. Although activity timing plays a key role in information processing, this temporal patterning has never been explored across the lateral bulb itself, although the temporal differences between the medial and lateral areas of the OB have been investigated using multichannel recordings. This study highlighted important temporal response differences across the OB that are associated with temporal activations at the epithelium [32].

Here, using a dual camera imaging approach for simultaneous recording of odor responses, we examined both the spatial and temporal odor patterning in the lOB in concert with the dOB and, in doing so, also uncover a novel mechanosensory response in the lateral bulb.

## Results

### Dual imaging of the dOB and lOB

**Fig 1:**
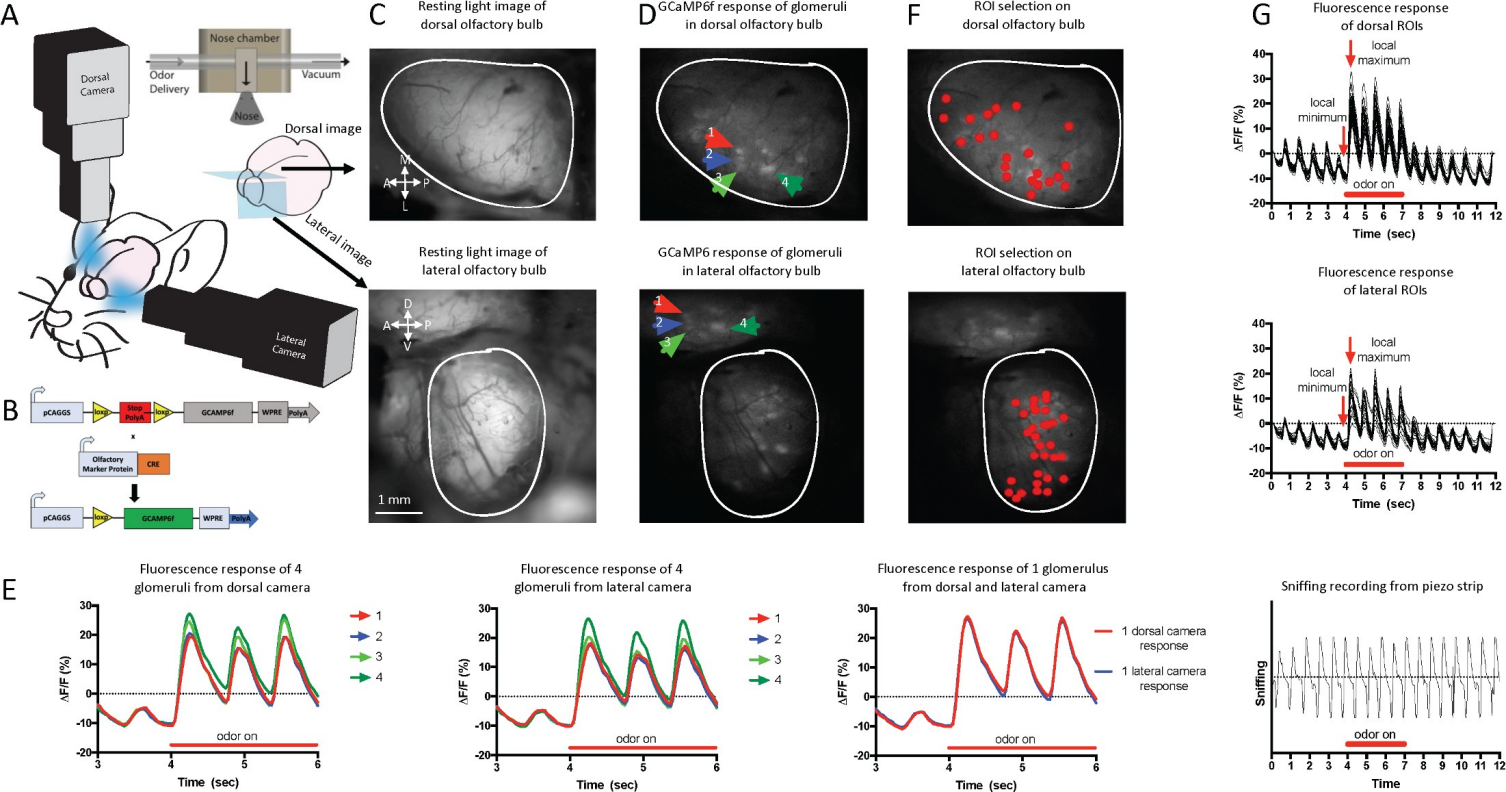
Dual imaging of the dOB and lOB in GCaMP6f reporter mice. (A) Schematic representation of the dual microscope imaging setup. Illustrating the plane of the dorsal and lateral images. Inset: schematic of the odor delivery assembly relative to the nostrils, illustrating the odor and vacuum flow channels. (B) Schematic of the mouse genotype. (C) Top: resting light image of the dOB. Bottom: resting light image of the lateral region of the OB. (D) Top: odor-induced activation map of the dOB. Arrows highlight 4 individual glomeruli. Bottom: odor-induced activation map of the lOB. Arrows point to the same 4 glomeruli as top. (E) Left: glomerular response traces from the 4 glomeruli imaged with the dorsal camera in response to 1% heptanone. Middle: responses from the 4 glomeruli imaged with the lateral camera in response to 1% heptanone. Right: glomerulus 4 imaged from the dorsal and lateral camera, overlaid. (F) Top: an example of the ROI selection on the dorsal bulb for 1 animal. Bottom: an example of the ROI selection for the lateral bulb for 1 animal. (G) Top: % ΔF/F responses for the dorsal ROIs in panel F (top) selection of first odor response is indicated (1 animal). Middle: % ΔF/F responses for the lateral ROIs in panel F (Bottom) (1 animal). Bottom: a sniffing trace from the same animal. Inhalation is the upward inflection (see Methods for details). Underlying data for this figure can be found in S1 Data. dOB, dorsal olfactory bulb; lOB, lateral olfactory bulb; OB, olfactory bulb; ROI, region of interest.

Here, we have developed a dual imaging approach (Fig 1A) to measure the glomerular activity of both the dorsal and lateral regions of the OB. We investigated OR sensory neuron input using *floxed-GCaMP6f* reporter mice crossed with *OMP-Cre* animals [12] (Fig 1B). In addition to implementing classical dorsal region windowing of the OB (Fig 1C top), we also successfully exposed the lateral region of the OB by unilateral enucleation and preparation of an optical window medial to the eye (Fig 1C bottom). Both cameras were synchronized for simultaneous fluorescent imaging of these two regions of the OB. These two orthogonal imaging macroscopes had overlapping imaging planes in the lateral region of the dOB (Fig 1D). Four glomeruli confirmed excellent agreement in the glomerular fluorescence responses imaged by the dorsal and lateral camera (Fig 1E). Using frame subtraction before and after the first odor inhalation, regions of interest (ROIs) were accumulated (i.e., an ROI that responds to at least one stimulus) across odors in both the lateral and dorsal images (Fig 1F), and their fluorescence (% ΔF/F) response traces were isolated and analyzed (Fig 1G). We examined approximately 1.5 times the number of glomeruli in the dOB as in the lOB (dorsal: 232, lateral: 149 across 6 animals; S1 Table). For the first time, we simultaneously imaged OSN calcium activity in the dorsal (dOB) and lateral (lOB) regions of the OB.

### Simultaneous odor mapping of the dOB and lOB

**Fig 2:**
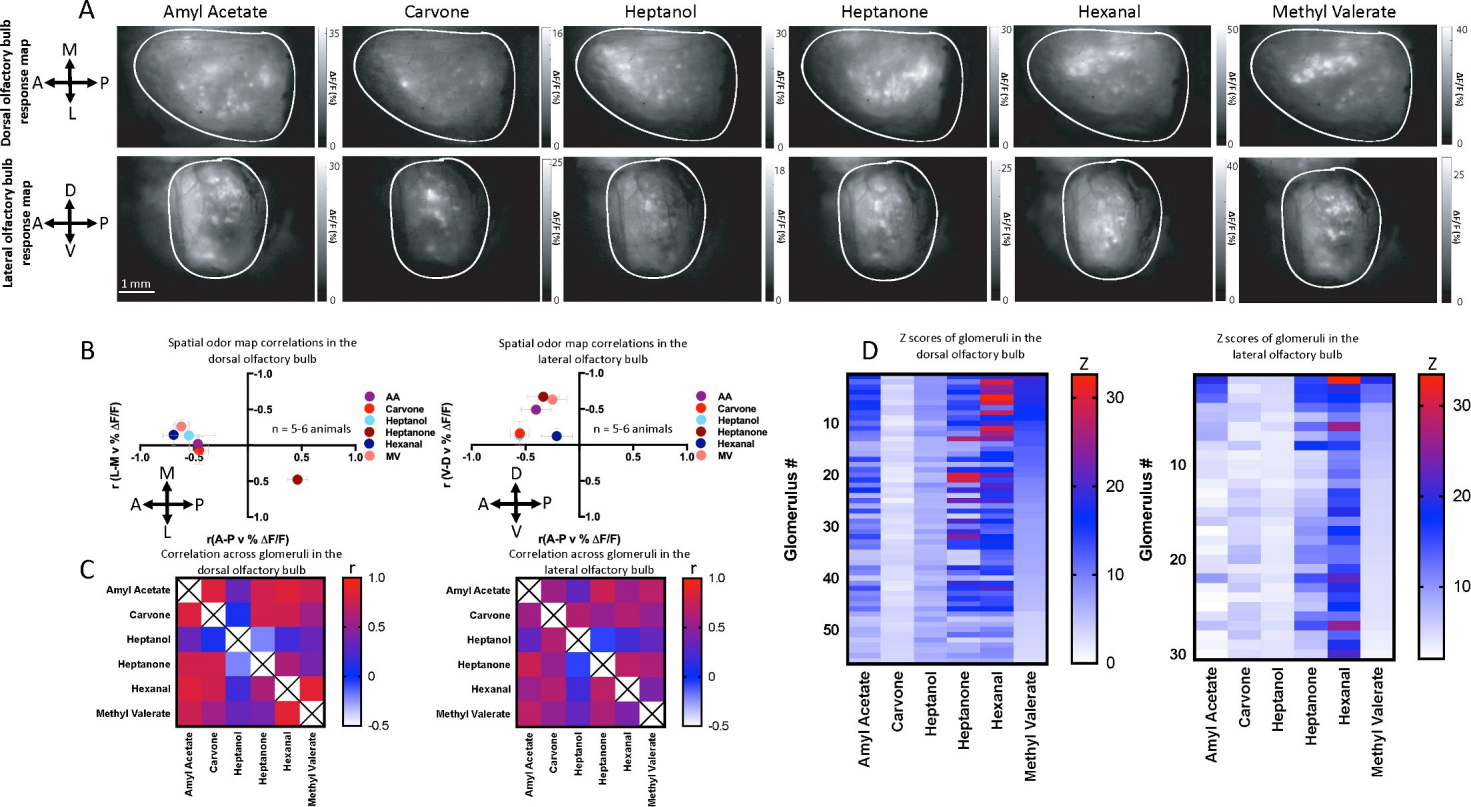
Simultaneous odor mapping of the dOB and lOB. (A) Odor-induced activation maps for AA, carvone, heptanol, heptanone, hexanal, and MV. Top row: the dorsal region of the OB. Bottom row: the lateral region of the OB (1 animal, average of 3 trials). (B) Spatial odor map correlations between response amplitudes and the location along each spatial dimension of all identified glomeruli. Left: dorsal region of the OB. Right: the lateral region of the OB. Error bars are SEM (AA, heptanol, and hexanal 5 animals; all others 6 animals). (C) Across-glomerular response pattern similarities, assessed by Pearson correlations across glomerular response amplitudes. Left: for the dOB. Right: for the lOB (AA, heptanol, and hexanal 5 animals; all others 6 animals). (D) Average z-scores of glomerular response amplitudes (relative to pre-odor breathing response amplitudes). z-Scores are organized relative to MV. Left represents the dorsal region of the OB. Right: the lateral region of the OB (1 animal, 3 trials, same trials as shown in 2A). Underlying data for this figure can be found in S1 Data. AA, amyl acetate; dOB, dorsal olfactory bulb; lOB, lateral olfactory bulb; MV, methyl valerate; OB, olfactory bulb.

OSNs expressing different receptors that recognize related odor molecules project to neighboring glomeruli in the OB [33]. Therefore, there is chemotopic organization of the glomerular responses represented in spatial odor patterns [10, 34]. Differences in the spatial activation patterns of glomeruli are thought to play a primary role in identifying odors [10, 35]. Using our dual imaging approach, we investigated spatial odor patterns of glomerular OSN input (*OMP-GCaMP6f* mice) responses over the first respiration cycle in both the dOB and lOB (Fig 2A 1 animal, 3 trials). Six odors—amyl acetate (AA), carvone, heptanol, heptanone, hexanal, and methyl valerate (MV)—at 1% (saturated vapor [s.v.]) concentration were used. These odors were chosen to represent different molecular groups, as well as to be represented in the lateral bulb (though without concern where in the lOB) [10, 21, 24]. The global spatial organization of activation of glomeruli in the anterio-posterior (A-P), dorso-ventral (D-V), or medio-lateral (M-L) dimensions was determined using linear correlations of glomerular response amplitudes with their spatial location (see Methods and S1A Fig for examples). All these analyses were based on images of 512 × 512 pixels, where lower pixel numbers along the x-axis represent more anterior regions in the dOB and lOB. A fixed set of ROIs, used in every analysis, was accumulated across odor maps for each mouse, where each ROI responded to at least one odorant. For laterality of the dOB, a low pixel number along the y-axis represented more medial locations of ROIs. Each hemi-bulb was analyzed separately, and only the hemi-bulb ipsilateral to the exposed lOB is shown here. For the lOB, a lower y-axis pixel number represented more dorsal regions. Correlation analysis of glomerular responses with spatial location provides a simple metric that highlighted the differences in global response patterns for each odor (%ΔF/F responses of all odors shown in S2 Fig).

In the dOB, all odors except heptanone clustered around intermediate anterior and intermediate mid-lateral locations. Correlations of heptanone glomerular response amplitudes with their location along the A-P dimension demonstrate a posteriorly dominated OB response. AA and carvone had no clear dominance along the M-L dimension. Heptanone was most dominant in the lateral dOB and MV in the medial region of the dOB (Fig 2B left, *n* = 5–6 animals, S2A Table). The odorants chosen for this study consist of a range of key molecular features, and the activation patterns of glomeruli for these odors differed across the dOB in both the A-P and M-L dimension.

Glomerular odor patterns in the lOB were all dominant in the dorsal and anterior regions, thus showing similar coarse spatial organization, in contrast to the differences in their spatial patterns of the dOB (Fig 2B right, *n* = 5–6 animals, S2B Table). No odor dominated the ventral or posterior region of the lOB. The contrasting activation patterns of these odorants between the dOB and lOB suggest that the receptors within the epithelial zones may be relaying different chemical information for the same odor.

We have highlighted the different spatial activation patterns across odors; however, their patterns can be similar in glomerular activation as ORs can transduce multiple odors [7]. We further investigated the correlations of glomerular responses across odors within the dOB (Fig 2C left, *n* = 5–6 animals) and the lOB (Fig 2C right, *n* = 5–6 animals), irrespective of location (the across-glomerular response patterns). These correlation matrices looked rather similar in general, in particular low correlations with heptanol response patterns (−0.21 to 0.34 and 0.64) amid otherwise mostly intermediate-high correlations, but some differences were clear. Glomerular activation was strong in the posterior region of the dOB in response to heptanone and strong in the anterior region for hexanal. Nevertheless, fairly high correlations between their glomerular response patterns highlighted that these odors activate similar glomeruli, suggesting that similarities in their odor structure are also being conveyed across the dOB (S3A Table). AA highly correlated with all odors except heptanol, demonstrating that a large number of the same glomeruli are being activated across odors.

In the lateral bulb, there was a narrower range of correlations between glomerular activations across odors (r = −0.06 to 0.74; S3B Table) than in the dOB (r = −0.21 to 0.86). The most similar response patterns in the dOB (AA-MV, r = 0.86) were much less similar in the lOB (r = 0.41, Fig 2C right). The two odors with a weak negative correlation in the dOB (heptanone and heptanol) are also weakly negatively correlated in the lOB. In contrast, carvone and heptanol are weakly correlated in the dOB and more correlated in the lOB. The degree of correlation similarity between the dOB and lOB appears to be odor dependent and highlights differences in overlapping receptor activation patterns for these odors when comparing the dOB (zone 1) and the lOB (zone 2–4).

To compare the response amplitudes of individual glomeruli across odors, the responses were z-scored relative to the standard deviation (SD) and mean of the pre-odor breathing responses. Glomerular responses in Fig 2A were organized relative to MV from high to low z-scored odor responses for both the dOB (Fig 2D left, *n* = 1 animal, 3 trials) and lOB (Fig 2D right, *n* = 1 animal, 3 trials). This demonstrates many highly significant odor responses (z > 3) and further shows the basis of the correlations of the odor responses (Fig 2C), highlighting the similarity in glomerular activation patterns across odors. We have shown here the distinct spatial odor patterns of the dOB and lOB, where their different chemotopy offers an insight into the integration of OR activation across all epithelial zones.

### Dorsal and lateral glomeruli response dynamics

Glomerular activation can evolve during a single odor sniff cycle [19, 36], and temporal glomerular responses can be used to facilitate odor coding [37]. The temporal dynamics of the glomerular responses within the first sniff after odor delivery were determined using T90 values (Methods and S1B Fig). T90 is the time from the start of inhalation to 90% of the peak amplitude. T90 was based on a double sigmoidal fit and is more robust than T50 and T20, due to the lower impact of sampling jitter.

Orthonasal airflow within the nasal cavity develops from the central domain of the dorsal meatus, which is associated with dOB projections, to the medial and lateral recesses of the ethmoid turbinates, having OSN projections to the lOB [38]. We examined the temporal activation differences between the dOB and lOB across odors using average T90 responses from all glomeruli (S2A and S2B Fig). Temporal response latencies differed strongly across odors (*P* < 0.0001, F(5, 2108) = 131.1), which also varied by region (*P* < 0.0001, F(5, 2108) = 6.26; interaction). A small but significant difference was observed in the overall temporal responses between the dOB and the lOB (*P* < 0.0024; F(1, 2108) = 9.22; two-way ANOVA [odor × OB region]) (S1C Fig).

In addition to average temporal responses, we also examined the temporal patterning across the OB spatial dimensions. The spatiotemporal dynamics of dorsal glomerular activation after odor presentation has a stereotypical progression from the posterior region of the dOB to the anterior region [19, 39]. We used linear correlations of T90 of glomeruli with their location along each spatial dimension (as performed in Fig 2B for response amplitude, here T90) to examine the global spatial organization of T90 of all the glomeruli. This spatiotemporal correlation analysis was performed on both the pre-odor response (breathing clean air) and the first sniff after odor presentation. This analysis would highlight any temporal dynamics associated with the mechanosensation of breathing compared to the odor delivered (S5A and S5B Table).

**Fig 3:**
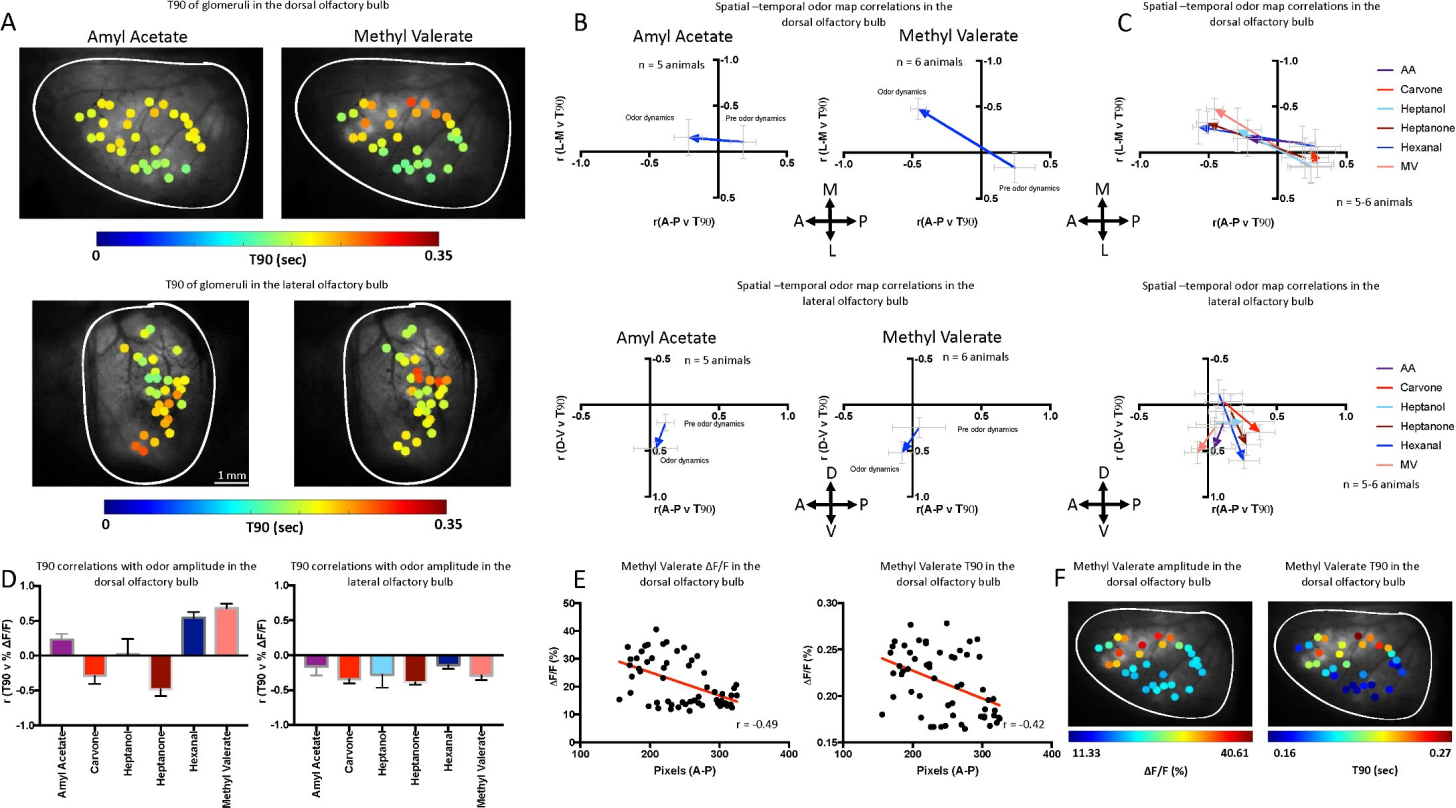
Temporal dynamics of the dOB and lOB. (A) Color-scaled T90 of responses by ROIs for AA and MV. Top: the dorsal region of the OB. Bottom: the lateral region of the OB (1 animal). (B) Comparison of spatiotemporal odor dynamics, i.e., correlation between T90s of glomeruli and their location along each dimension across the dOB of AA (*n* = 5 mice) and MV (*n* = 6 mice) from pre-odor (breathing response to clean air) to odor onset (1st odor response peak). Arrow represents the direction from pre-odor to odor on. Top: the dorsal region of the OB, data are represented in both the A-P directions and the M-L as shown in panel A. Bottom: the lateral region of the OB, data are represented in both the A-P directions and the D-V as shown in panel A. Error bars are SEM. (C) Spatiotemporal odor map dynamics for all odors. Top: the dorsal region of the OB. Bottom: the lateral region of the OB (AA, heptanol, and hexanal 5 animals; all others 6 animals). (D) T90 correlations with odor amplitude (ΔF/F). Left: the dorsal region of the OB. Right: the lateral region of the OB. Error bars are SEM (AA, heptanol, and hexanal 5 animals; all others 6 animals). (E) Left: correlation of glomerular response amplitudes to MV sorted from anterior to posterior in the dOB (r^2^ = 0.24, slope = −0.08 ± 0.02, 1 animal, 57 glomeruli). Right: correlation of glomerular T90 responses to MV sorted from anterior to posterior in the dOB (r^2^ = 0.18, slope = −0.0002 ± 0.0009, 1 animal, 57 glomeruli). (F) Left: amplitude of glomeruli represented on a color scale. Red indicates high responses and blue low. Right: T90 of glomeruli represented on a color scale. Red indicates the slowest responses and blue the fastest (1 animal [same as panel E], 57 glomeruli). Underlying data for this figure can be found in S1 Data. A-P, anterio-posterior; AA, amyl acetate; D-V, dorso-ventral; dOB, dorsal olfactory bulb; lOB, lateral olfactory bulb; M-L, medio-lateral; MV, methyl valerate; OB, olfactory bulb; ROI, region of interest.

AA and MV temporal patterns differed across dorsal glomeruli (Fig 3A, 1 animal, 3 trials, all odors are shown in S2 and S3 Figs). For both odors, the pre-odor temporal dynamics were in the posterior region of the dOB. Upon AA presentation, the spatiotemporal dynamics shift from posterior to anterior (pre odor versus odor: A-P *P* = 0.03, M-L *P* = 0.80, *n* = 5 mice) (Fig 3B left). Upon MV presentation, spatiotemporal dynamics showed a larger shift (Fig 3B right) (pre odor versus odor: A-P *P* = 0.0017, M-L *P* = 0.01, *n* = 6 mice) with faster responses at the posterior-lateral region and slower responses (higher T90 responses) at the anterior-medial region. Spatiotemporal dynamics were slower in the anterior-medial region for 5 of the 6 odors (Fig 3C). T90 responses in the posterior dOB and the anterior dOB strongly depend on dOB region (*P* < 0.0001, F(1, 1278) = 144.5) and odor (*P* < 0.0001, F(5, 1278) = 141.1; two-way ANOVA [odor × dOB region]) (S1D Fig).

In the lOB, slower responses were observed in the ventral region for both AA and MV (pre odor versus odor: A-P *P* = 0.68, D-V *P* = 0.11, *n* = 5 mice; pre odor versus odor: A-P *P* = 0.57, D-V *P* = 0.05, *n* = 6 mice) (Fig 3A and Fig 3B bottom). Spatiotemporal dynamics were slower in the ventral region for 5 of the 6 odors (Fig 3C). Similar to the dOB, the responses in the dorsal and ventral lOB are strongly influenced by region (*P* < 0.0001, F(1, 818) = 91.33) and odor *(P* < 0.0001, F(5, 818) = 40.67; two-way ANOVA [odor × lOB region]) (S1E Fig).

The spatiotemporal dynamics of the lOB odor responses, but not clean air breathing responses, hence progress in the D-V pattern whereby A-P shift was odor dependent. These were very different from the posterior-lateral to anterior-medial odor evoked dynamics simultaneously observed in the dOB. The dynamics are in line with the airflow progression throughout the nasal cavity and the corresponding zones within the bulb [15]. The spatiotemporal dynamics were odor dependent and were not present in pre-odor inhalation-based (mechanosensory) dynamics.

Correlation analysis of odor response amplitudes and T90s across glomeruli, averaged across 5–6 mice, suggests that the spatiotemporal patterns were also not predictable by the response amplitude of each odor in the dOB (Fig 3D left, S6 Table). This was in line with previous studies that have demonstrated that response latencies are not exclusively determined by the response amplitude of the glomeruli [19, 39]. However, in the lOB odor response, amplitudes and T90s were negatively correlated for all odors (Fig 3D Right, S6 Table), suggesting that their interaction is predominant in the lOB (though noting absence of strong responses ventrally). The spatial representations of all odors in the lOB were tightly clustered in the dorsal region and thus consistently negatively correlated with T90.

To show that stronger responses do not by necessity result in faster responses, as appears to be the case for the lOB (Fig 3D), we show in Fig 3E and 3F that, for MV, the highest response amplitudes were in the anterior region of the dOB, and this was also the region of slowest responses (1 animal, 3 trials, 57 glomeruli). We demonstrate here that the spatiotemporal dynamics emerge in the dOB and lOB only as a result of odor stimulation, yet this is not a meta-effect of OSN odor response amplitude nor mechanosensation per se. The temporal dynamics of the lOB differ from those of the dOB and may provide additional temporal information relating to the OR projections from the nasal cavity and the airflow and sorption patterns of odor intake.

### Glomerular odor concentration dependence

**Fig 4:**
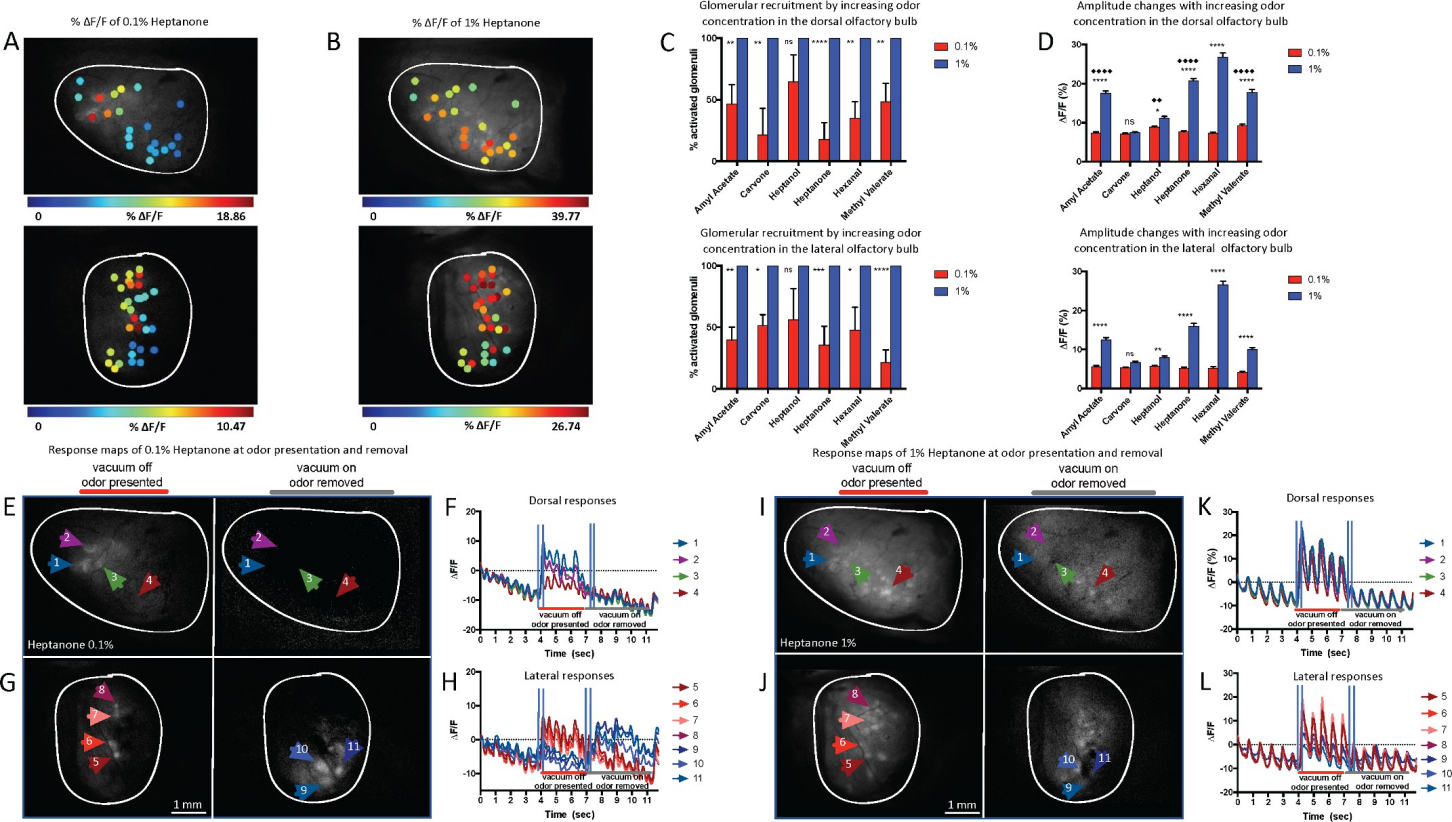
Glomerular odor concentration dependence. (A) Color-scaled response amplitudes for chosen ROIs for 0.1% heptanone. Top: the dorsal region of the OB. Bottom: the lateral region of the OB. (B) Color-scaled responses amplitude for chosen ROIs for 1% heptanone. Top: the dorsal region of the OB. Bottom: the lateral region of the OB. (C) Percent of glomeruli that are significantly activated by 0.1% (AA, heptanol, hexanal, and MV 5 animals; all others 6 animals) compared to the higher concentration of 1% (AA, heptanol, and hexanal 5 animals; all others 6 animals). The number of responding glomeruli to the higher odor concentration has been normalized to 100%. Top: the dorsal region of the OB. Bottom: the lateral region of the OB. Statistics represent two-way ANOVA (odor × concentration) per OB region with Bonferroni’s multiple comparisons test for concentration effect. Error bars are SEM. *P < 0.05, **P < 0.01, ***P <0.001, ****P < 0.0001. (D) Average ΔF/F responses for odors comparing 0.1% and 1% concentration. Top: the dorsal region of the OB. Bottom: the lateral region of the OB. Statistics as in panel C. Additional statistics represent two-way ANOVA (odor × OB region) with Bonferroni’s multiple comparisons test for OB region effect at 1% s.v. ♦*P* < 0.05, ♦♦*P* < 0.01, ♦♦♦*P* <0.001, ♦♦♦♦*P* < 0.0001. (E) Odor-induced activation maps for 0.1% heptanone presentation in the dorsal region of the OB, arrows indicate 4 glomeruli. Left: during odor presentation. Right: after the odor is removed by a vacuum. Arrows indicate the same glomeruli as left. (F) Glomerular response traces for the 4 glomeruli in panel E. Vertical blue bars indicate response and reference frames to compute response maps. (G) Odor-induced activation maps for 0.1% heptanone presentation in the lateral region of the OB, arrows indicate 7 glomeruli. Left: during odor presentation. Arrows show 4 of the glomeruli. Right: after the end of odor presentation. Arrows represent 3 additional glomeruli. (H) Glomerular response traces for the 7 glomeruli in panel F. Glomeruli 5–8 (reds) respond during odor presentation, and glomeruli 9–11 respond when the odor is removed by vacuum (blues). I–L same as E–H but for 1% heptanone. Underlying data for this figure can be found in S1 Data. AA, amyl acetate; MV, metyl valerate; OB, olfactory bulb; ROI, region of interest.

At low odor concentrations, only ORs with the highest affinity for a given odor will respond. With increasing odor concentration, additional glomeruli with lower affinity for the odor are activated [12, 39]. This recruitment of glomeruli for OSN input in response to odor concentration has been widely studied in the dOB, but not in lOB [12, 16, 25, 40]. Therefore, we explored the level of glomerular recruitment in the lOB. We compared the glomerular response patterns of two odor concentrations (0.1% and 1% [s.v.]) (Fig 4A and 4B, blue dots indicating low response amplitude and red dots high response amplitudes). Recruitment of glomeruli by higher odor concentration (Fig 4C top) was determined by whether the response amplitude to the first odor sniff was significantly above pre-odor breathing response amplitudes (*P* < 0.01). The number of glomeruli activated by the 1% odor concentration was normalized to 100%, and we report for the 0.1% concentration the number of activated glomeruli relative to 1% s.v. (S7 Table).

We confirmed that the higher odor concentration recruited additional glomeruli in both the dOB (*P* < 0.0001, F (1, 46) = 79.68, two-way ANOVA [odor × concentration]) and the lOB (*P* < 0.0001, F (1, 50) = 80.14). Recruitment did not consistently differ across odors (*P* = 0.43, F(5, 48) = 0.97, two-way ANOVA [odor × OB region]) or between the lOB and dOB (*P* = 0.74, F(1, 48) = 0.11) nor did it show a significant interaction between odor and OB region (*P* = 0.54, F(5, 48) = 0.81).

We confirmed also that the higher odor concentration evoked stronger glomerular responses in both the dOB (*P* < 0.0001, F (1, 2540) = 929, two-way ANOVA [odor × concentration]) and the lOB (*P* < 0.0001, F (1, 1638) = 923.1), which depended on the odor (interaction: *P* < 0.0001, F (5, 2540) = 95.26 and *P* < 0.0001, F (5, 1638) = 123.5, respectively). Average response amplitudes of glomeruli were significantly larger in the dOB than lOB across all odors at 1% s.v. (*P* < 0.0001, F(1, 2108) = 89.32, two-way ANOVA [odor × OB region]; S8 Table), suggesting the dOB is generally more responsive than the lOB (Fig 4D). Amplitude significantly differed across odors (*P* < 0.0001, F(5, 2108) = 215), and the interaction between odor and region was significant (*P* < 0.0001, F(5, 2108) = 9.45).

For 0.1% concentration, a small subset of glomeruli in the ventral region of the lOB responded after odor delivery. This activation of glomeruli after odor delivery was not observed in the dOB. We explored 4 glomeruli in response to 0.1% heptanone in the dOB (Fig 4E) alongside the %ΔF/F responses of these glomeruli (Fig 4F). In the lOB, we examined 7 glomeruli (Fig 4G) of which 4 responded to the odor and 3 responded post odor (Fig 4H). This phenomenon was not observed in trials using the higher 1% odor concentration. The same glomeruli were compared (Fig 4I and 4J), and no activation post odor was observed (Fig 4K and 4L). Comparison of all glomeruli across the 6 odors in the lOB determined that during trials presenting 0.1% odor, 50.6% of glomeruli (*n* = 149 glomeruli) had a higher amplitude in response to odor removal (just after vacuum onset) than to odor onset (just after vacuum was turned off). For 1% odor trials, only 9.6% of these glomeruli (*n* = 149 glomeruli) had higher amplitude response to odor removal than to odor onset. This demonstrates a subset of glomeruli that respond post odor primarily in low concentration presentations.

### The lOB displays unique mechanosensitive activation

Previous studies have demonstrated that OSNs can sense two modalities, chemical and mechanical. Mechanical stimulation enhances the response of OSNs to weak stimulation of odorants [41, 42], and a loss of mechanosensation impairs phase coding in mitral/tufted cells [43]. During low odor concentrations, individual glomeruli in the lateral bulb were responsive at two key points during the trial, namely, during odor delivery and post odor during removal by a vacuum (Fig 4E–4H).

**Fig 5:**
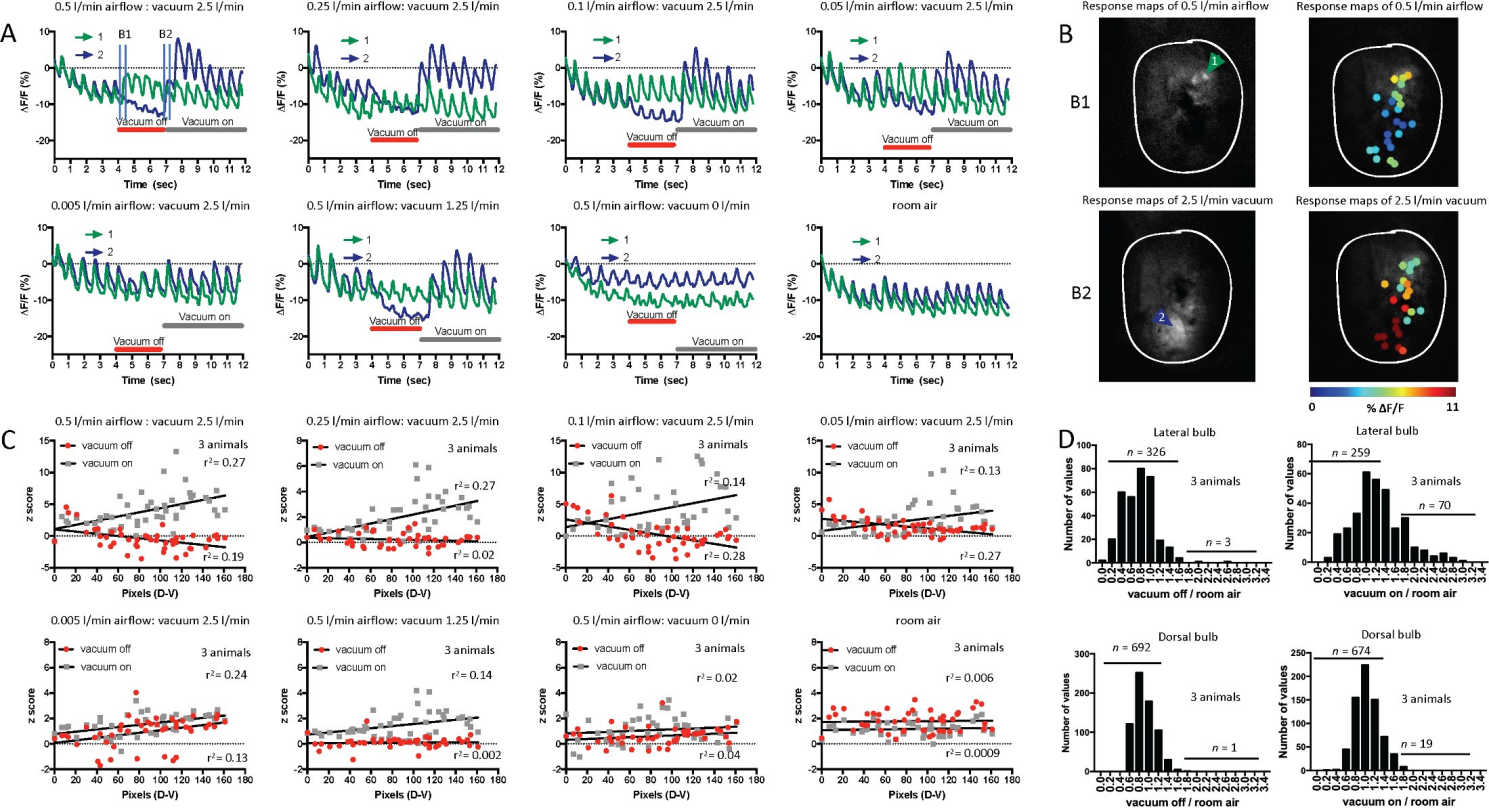
The lOB displays mechanosensitive activation beyond breathing. (A) Raw traces (% ΔF/F) of two glomerular breathing responses during presentation of clean air flow rates (0.5, 0.25, 0.1, 0.05, and 0.005 L/min) and vacuum rates (2.5, 1.25, and 0 L/min) and room air with no external flows (1 animal, 2 glomeruli). These are presented as examples of the trial stimulus; green represents a glomerulus in the dorsal region of the lOB, blue in the ventral (maps shown in panel B). Red bar illustrates the period in which only the air is presented (“Vacuum off”), and grey is when the vacuum is also turned on (“Vacuum on”); height indicates flow rates relative to other trials. Vertical blue bars indicate response and reference frames to compute response maps. (B1, vacuum off response; B2, vacuum on response; maps shown in panel B). (B) Activation map in response to inspiration during only clean air flow rate of 0.5 L/min (Left top) and additionally the vacuum 2.5 L/min (Left bottom). Arrows indicate the ROI displayed in panel A. B1 and B2 indicate the respective time points in panel A (0.5 L/min airflow: vacuum 2.5 L/min). Right: activation map of all glomeruli chosen to clean air flow rate of 0.5 L/min (Top) and vacuum 2.5 L/min (Bottom). (C) The z-scores of the lOB glomerular responses during only clean air flow (red) and also vacuum (grey), organized from dorsal to ventral, for different clean air flow rates (0.5, 0.25, 0.1, 0.05, and 0.005 L/min) and vacuum rates (2.5, 1.25, and 0 L/min) and room air (3 animals, 47 glomeruli). Linear correlation fits are indicated. (D) Top left: histogram of the ratio of vacuum off responses relative to room air breathing responses in the lateral bulb. Top right: histogram of the ratio of vacuum on breathing responses relative to room air breathing responses in the lateral bulb (3 animals, 47 glomeruli, total 329 glomerular responses across all flow rates). Number of responses (*n*) was divided into number of glomerular responses <1.6 and >1.6 times above the room air breathing response. Bottom: same as top but in the dorsal bulb. Underlying data for this figure can be found in S1 Data. lOB, lateral olfactory bulb; OB, olfactory bulb; ROI, region of interest.

We next investigated whether this glomerular response to post–odor-vacuum onset was due to odor removal (change in chemical environment) or due to air-flow–related pressure change (mechanosensation). We replaced odor delivery with clean air and used the same vacuum for air removal in this subexperiment in an additional 3 mice. From these trials, we show the fluorescent response (% ΔF/F) for two glomeruli (Fig 5A) over the 12-s trial period during air presentation and vacuum. One glomerulus was chosen from the dorsal region and one from the ventral region of the lOB (Fig 5B), a region we previously showed to be responsive to vacuum (Fig 4G and 4H). During the initial 0.5 L/min air delivery and 2.5 L/min vacuum period, both glomeruli displayed stereotypical breathing responses (Fig 5A) [44]. These were mostly absent in the ventral glomerulus during air delivery (i.e., vacuum off), while the dorsal glomerulus remained unchanged. When the vacuum flow was reintroduced, the ventral glomerulus was strongly activated. We investigated a range of clean air flow rates (0.5, 0.25, 0.1, 0.05, and 0.005 L/min) and vacuum flow rates (2.5, 1.25, and 0 L/min) to examine whether they were affecting the glomerular responses. The dorsal glomerulus remained unresponsive across clean air flow rates; however, the ventral glomerulus amplitude response changed with both the air and vacuum flow rate (Fig 5A).

The z-scores of glomerular responses to both air and vacuum across the D-V spatial axis (Fig 5C) were used to examine whether there was a spatial organization of the lOB glomerular activation. During airflow (0.5 L/min), glomerular responses did not show a D-V dominance; however, during vacuum (2.5 L/min), large responses consistently corresponded with the ventral region of the lOB (S9 Table). When the air (0.005 L/min) or vacuum (0 L/min) flow rates were lowest, the responses of the ventral glomeruli were similar to that observed when the trial stimulus was breathing room air (“room air”). This was surprising, as their respective vacuum (2.5 L/min) and airflow (0.5 L/min) rates were high. This suggests that it was not a single stimulus that the ventral glomerulus is responding to but some nonlinear combination of both the clean air and vacuum flow rates. We show the equivalent data for the dOB in S5 Fig, where it is clear that dOB glomeruli lack this sensitivity.

The magnitude of the glomerular responses relative to breathing room air was determined across all stimulus conditions (Fig 5D). During clean air presentation, most lOB glomeruli had a response below breathing amplitudes. However, during vacuum, there was a bimodal distribution of glomerular responses with an initial distribution similar to air, and an additional distribution up to 3 times the room air breathing response. This was not observed in the dOB.

**Fig 6:**
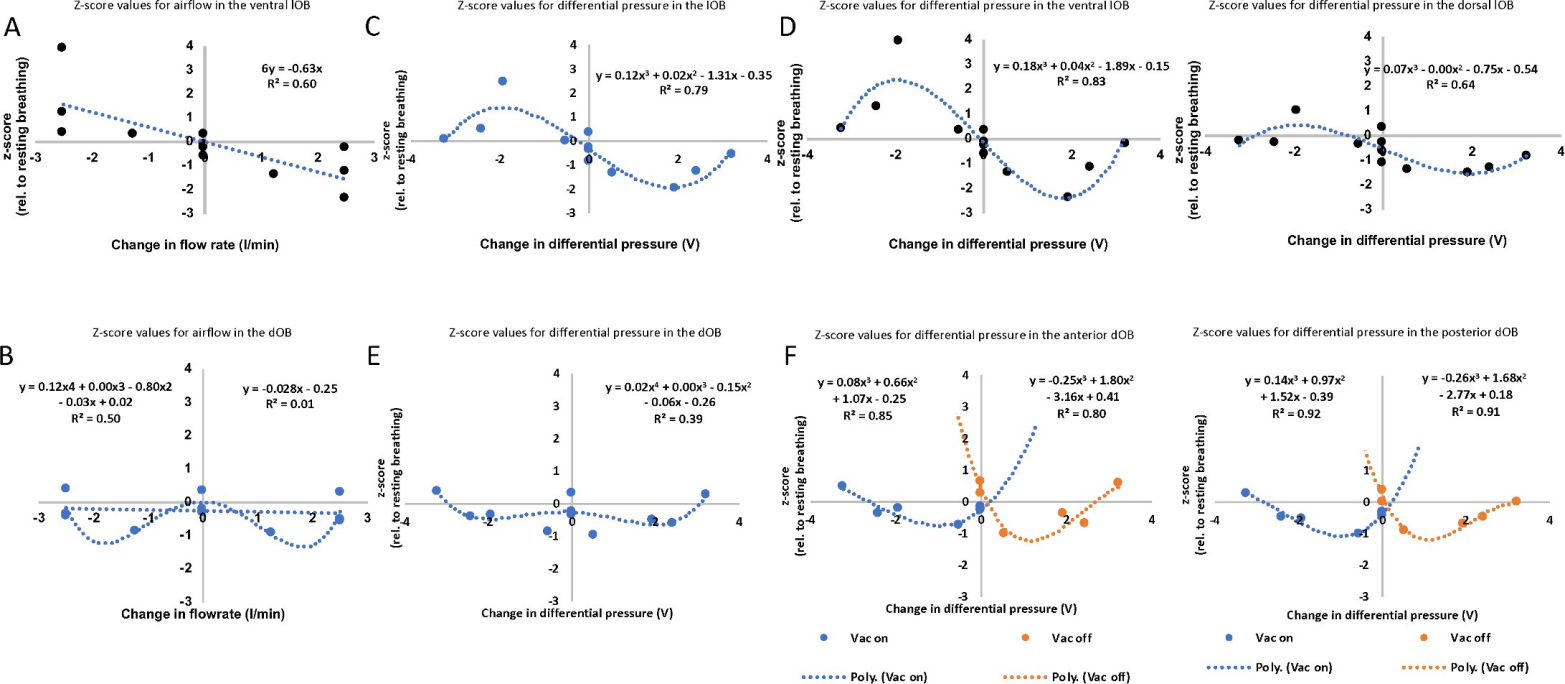
The lOB is sensitive to differential pressure. (A) Mean z-scored OB responses to different flow rates in the ventral lOB. (B) Mean z-scored OB responses to different flow rates in the dOB. (C) Mean z-scored OB responses to different pressure changes in the lOB. (D) Mean z-scored OB responses to different pressure changes in the lOB. Left: the ventral lOB. Right: the dorsal lOB. (E) Mean z-scored OB responses to different pressure changes in the dOB. (F) Mean z-scored OB responses to different pressure changes in the dOB. Left: the anterior dOB. Right: the posterior dOB: 3 animals; dOB: 99 glomeruli; and lOB: 47 glomeruli. Graphs are reorganized from S4C and S4D Fig, all offset by respective z-scores for no–flow-change condition (0–0, room; see Methods). Only the lOB—and the vlOB in particular—shows a strong and positive response to an intermediary drop in pressure at the odor delivery tube. The dOB only shows mild response suppression, particularly by intermediate changes in pressure. Pressure unit is relative only. Underlying data for this figure can be found in S1 Data. dOB, dorsal olfactory bulb; lOB, lateral olfactory bulb; OB, olfactory bulb; vlOB,

We next examined whether this response was related to flow rate or the change in pressure. Even though the flow rates are similar, the differential pressure change is not (S4A and S4B Fig), and therefore the flow rates cannot explain the consistently varying responses (Fig 6A). The vast difference in responsiveness of dOB and lOB glomeruli to these stimuli is clearly shown in S4C and S4D Fig. We show that the lOB responds positively to a negative pressure change (vacuum on) and negatively to a positive pressure change (vacuum off) (Fig 6C) primarily in the ventral lOB and more weakly in the dorsal lOB (Fig 6D). This, however, was not observed in the dOB (Fig 6E and 6F). In the lOB, a single third-order polynomial can accurately capture the glomerular responses across pressure change values, due to the large uneven order terms to capture the response asymmetry around x = 0. In the dOB, however, the responses can only adequately be captured by a separate fit for the negative and positive pressure changes. Our results show a unique set of glomeruli in the ventral region of the lOB that respond to the change in pressure of air and vacuum, a phenomenon not previously observed.

## Discussion

Here, we explore both the spatial and temporal patterning of both the chemosensory and mechanosensory input responses to the lOB and dOB in tandem for the first time. This is achieved through simultaneous imaging and expands upon the spatial and temporal information that has been extensively gleaned only from the dOB. We have not only examined how odor information is presented at the lOB but also how this compares to the dOB. In addition, we have also uncovered a unique mechanosensory response—unlike breathing—that is, interestingly, completely unique to the lOB.

Spatial and temporal patterning in the OB is influenced by a number of factors such as the molecular features of the odor that enters the nasal cavity [15], the OR distribution across the olfactory epithelium (OE) [5], and the sorption of the odor into the mucus layer of the nose [13, 45, 46]. The spatial patterns of odors have been excellently described throughout the entire glomerular layer using 2-DG [20, 21, 47] and fMRI [11]. Although high–spatial-resolution maps using intrinsic and calcium imaging have primarily focused on the dOB [25, 40, 48], intrinsic imaging has previously been used to explore the lateral bulb [20, 21, 47]. Here, we expand this knowledge by investigating the presynaptic glomerular responses to odors with various molecular groups (ester, terpenoid, alcohol, ketone, alkyl aldehyde, methyl ester) simultaneously in the dOB and lOB, seamlessly evaluating a greater area of the glomerular sheet.

Previously, spatial odor maps of the lOB, visualized with intrinsic imaging, defined 2 key areas of activation: one in the dorsal region and one in the ventral region [20]. It was concluded that odorants carvone, hexanal, and heptanone all had primary activation in the more ventral region, which is contrasted by our data presented here. Differences between calcium and intrinsically imaged spatial odor maps have previously been observed [40], and comparisons of calcium and intrinsic imaging have highlighted the temporal response differences between the two methods. Firstly, the half-maximum time of an intrinsic response is up to 6 times slower than calcium, and secondly, intrinsic signals saturate quickly, potentially misrepresenting glomerular odor responses for other neuronal processes [23]. Using intrinsic imaging of the lOB, nearly all odors were mapped to the ventral region, which in our study is where we observe a mechanosensory response. It is possible that within that study, the same mechanosensory responses were being observed, which could not be resolved from odor activation because the technique lacked temporal resolution. Therefore, we suggest that this current study, affording a higher temporal resolution, more accurately represents the spatial odor patterns in the lOB.

Not only were we able to define the spatial odor patterns of the lOB, but we were also able to compare these to the dOB. Comparisons using the dual imaging approach has highlighted key differences between the spatial representations of odors in the dOB and lOB. For example, we highlighted that in the dOB the spatial maps of heptanone and MV were very different, while in the lOB they were very similar. On the dorsal surface, it is well established that odorants with similar molecular features group together [25, 40], and chemotopic organization has been shown in the lateral bulb [47]. Studies have also suggested that there only exists a coarse chemotopic organization with the dorsal bulb [49]. Carbon chain length has been shown to shift spatial maps in the dOB using intrinsic imaging [48]; however, using calcium imaging, little effect was observed [40]. Using fMRI, carbon chain length has been shown to shift spatial patterns in the lOB [11]. Previous spatial mapping of the lOB using intrinsic imaging has shown that the hydrocarbon skeleton is represented in the lOB [20]. In our case, heptanone, a ketone, and MV, an ester, only differ by one carbon in length. In line with these suggestions, the spatial maps of heptanone and MV may differ greatly dorsally because of differing functional groups but map similarly laterally because their carbon chain length is similar, although we admit our odor array is neither sufficiently large nor systematically diverse to strongly address the issue of chemotopy. Thus, the integration of information across the epithelium zones may dramatically increase the amount of information processed for a given odor and the subsequent differences between odors. A key consideration is the zonal distribution of OR classes across the zones in the OE. Class 1 ORs are expressed dorsally in zone 1, Class II ORs in zones 1–4, and trace amine-associated receptors (TAARs) mostly dorsally but some ventrally [50, 51]. Zone 1 subsequently maps to the medial part of the dOB, the dorsal TAARs to the caudiomedial part of the dOB, and the Class II ORs in zone 1–4 to any of the other regions of the glomerular layer [50]. The lOB likely only contains axonal terminals from OSNs with Class II receptors which are organized such that more dorsal lOB glomeruli receive afferents from more medial zone 1 OE, versus more ventral lOB glomeruli from lateral zone 4 OE [52]. The odorants chosen here must activate some ORs from both classes to some extent as odor responses are observed in both the dOB and the lOB for all odors.

Odor information processing has strongly been linked to the perception of temporal information related to odor presentation. Firstly, the temporal responses of mitral/tufted cells have been shown to lock to the sniff cycle, and behaviorally mice can detect temporal optogenetic odor information down to 10 ms relative to the sniff cycle [30]. However, this is not the sole source of temporal information as optogenetic timing differences across glomeruli can be perceived down to 13 ms irrespective of sniff cycle [31]. Temporal patterning across the OB may be a result of zonation within the OE. OSN projections to the dOB and lOB express receptors in different locations within the nasal cavity. The comparison across both regions of the bulb affords us the ability to not only look at the OR activations from the dorsal recess but all areas of the epithelium throughout an inhalation. The dorsal recess experiences higher odor concentrations and air flows compared to other areas of the epithelium, which may contribute to differing temporal patterns [53]. Multichannel recordings have described different temporal responses between mirror glomeruli in the medial OB and lOB [32, 54]. The ventral-medial glomeruli respond faster than the D-L glomeruli, which in our study show the fastest responses of the lOB overall. We observed that the odor responses were slower in the lOB compared to the dOB in line with airflow patterns.

We examined the temporal patterning across glomeruli, and it is well established that the dOB displays stereotypical wave of glomerular responses from the posterior-lateral region to the anterior-medial region [19, 55]. We extend this knowledge by also examining the temporal responses in the lateral region. The lateral bulb glomerular responses temporally vary along the D-V axis rather than the roughly A-P axis that is observed in the dOB. Previous optical imaging studies have highlighted that glomerular temporal patterns are locked to respiration [39]. This temporal progression is in line with the sequence of airflow across the epithelium [38]. We found that this temporal pattern is absent in clean air respiration and is not consistently explained by regional differences in amplitude of the odors. We hence demonstrate that the lOB and dOB each have unique global temporal patterning. In situ hybridization mapping of OSN OR projections from the nasal epithelium to the bulb illustrates a D-V arrangement of glomeruli that correlates with the dorsomedial/ventrolateral axis in the epithelium [52], in line with the airflow patterns that develop in the nasal cavity [38]. Considering that the temporal differences in activation of glomeruli across the dOB can be perceived by mice [31], it remains an open question whether the difference in temporal activation in the lOB is providing additional timing information, although this is likely for complex naturalistic odor mixtures.

It has been demonstrated previously that OSNs have dual functions, responding to both chemical and mechanical stimuli [41, 42], suggesting that these cells are providing airflow information in addition to odor detection. Respiration has long been known to modulate bulbar activity [56, 57] and perhaps serves as a reference for the temporal activity of glomeruli in response to odor. Mice can detect odor stimulation relative to the phase of the sniff cycle [30, 58], and mechanosensation is important for phase coding of odor identity [43]. We have demonstrated that the ventral lOB displays unusual sensitivity to mechanosensation. Although it is possible that the artificial flow rates have created a pressure change that is not normally present in the nose, it is likely these responses may highlight a region of the bulb specifically monitoring pressure change in the nasal cavity.

We have considered the possibility that odorants could play a role in these responses, as the vacuum may have drawn room odorants over the nares. Indeed, c-fos experiments have highlighted the ventral lOB responses to urine [59, 60]. Furthermore, TAARs are exquisitely sensitive, and while most project dorsally, Taar 6, 7a, 7b, and 7d may project ventrally [50]. However, every precaution was taken to ensure that the imaging setup was very clean for imaging. A room vacuum was placed above the imaging setup to continually cycle room air to prevent the accumulation of odors, and a high-efficiency particulate air (HEPA) and active carbon filter was also running constantly in the room during imaging. Although we cannot eliminate a role for odor completely, if indeed these responses were odor responses, they would be present in all experiments with vacuum, which isn’t the case.

We observed this mechanosensitive response only when both airflow and vacuum are present and not determined by airflow but the differential pressure created between both. This suggests that the response is not simply a result of either positive or negative pressure within the nose but the transition point between the two as in respiration. It further suggests some inhibitory mechanism of odor transduction onto this mechanosensory phenomenon. The receptor projections to the ventral region of the lOB originate from zone 4 within the OE. Unilateral nares occlusion leads to an increase in expression level of ORs specifically in this zone, suggesting that the loss of mechanosensation may have changed their expression level [61]. Interestingly, high airflow rates in the nasal cavity enhances the sensitivity to low odor concentrations but not high odor concentrations [62]. The sorption of odors into the mucosal layer is also enhanced by flow rate [63]. We show that the temporal patterning of odor responses in the lOB is slowest toward the ventral region, and potentially this mechanosensory response in the ventral lOB is providing the reference point for respiratory phase transitions or playing a role in enhancing the sensitivity to low odor concentrations.

Overall, these data have expanded our knowledge about both the spatial and temporal information about odorants across the dorsal and lateral presynaptic glomerular layer and highlighted a unique mechanosensory response unique to the lateral bulb.

## Methods

### Ethics statement

All procedures were performed in accordance with protocols approved by the Pierce Animal Care and Use Committee (PACUC) JV1-2016 and JV1-2019. These procedures are in agreement with the National Institutes of Health Guide for the Care and Use of Laboratory Animals (8th edition).

### Surgery

Nine *OMP-GCaMP6f* mice (generated by crossing *OMP-Cre* [Jax Stock #006668] with *GCaMP6f* floxed transgenic mice [Jax Stock #024105]) aged 12 to 20 wk, both males and females, were used. They were anaesthetized using isoflurane (4% for induction, 1.5%–2.5% for maintenance). Anesthetic maintenance was monitored using the pedal withdrawal reflex and supplemented as needed. Core body temperature of the animal was maintained at approximately 37°C with a thermostatically controlled heating pad. Post surgery, the animals were placed in their home cage on a heating pad. Carprofen (5 mg/kg, sc) was administered prior to surgery and Buprenorphine (50 μg/kg, IM) at the start of surgery. Mice received supplemental carprofen 24 h post surgery, and weight was monitored for the duration of the experiment. Animals were placed in a stereotaxic holder, and the animals were prepared using aseptic procedures. For exposure of the dOB, the skin was removed, and the underlying bone was thinned using a dental drill. For exposure of the lOB, an enucleation of the left eye was performed, and the upper and lower eyelids were removed. The bone overlying the lateral portion of the OB was thinned. A seamless covering of cyanoacrylate was applied to both the dorsal and lateral windows at once. A head cap was secured using cyanoacrylate and dental cement for stability during imaging. Animals were given at least 24 hr of recovery post surgery before imaging to reduce any OB inflammation as a result of windowing.

### Imaging

All imaging was carried out between 24 and 72 hr post surgery after full recovery. Imaging was performed under ketamine:dexdomitor (100:0.5 mg/kg, i.p, 25% boosters). Atropine (0.03 mg/kg, i.p.) was administered at the start of imaging and every 2 hr hereafter. Eye lubricant was used throughout (Lubrifresh P.M. lubricant eye ointment).

Simultaneous recordings of the dOB and lOB were made using 2 identical setups consisting of 2 Hamamatsu ORCA Flash 4.0 LT sCMOS cameras (Hamamatsu, Japan) at a frame rate of 30 Hz and with 4 × 4 binning to 512 × 512 pixels. Two high-power LED 470 nm (Thorlabs, Newark, NJ) was driven by a T-Cube LED Driver (LEDD1B, Thorlabs, Newark, NJ). The custom-made tandem-lens type [64] was used at a 2.7 magnification (FOV: 5 × 5 mm). Imaging lenses were prime Nikon F-mount (ccd lens: 135 mm f/3.5, used at f/8; object lens: 50 mm f/3.5, used at f/3.5). Custom code written in LabView (National Instruments) controlled simultaneous image acquisition using both sCMOS cameras and timing controls for the light source and odor delivery. Sniffing and odor presentation data were acquired simultaneously through a National Instruments data acquisition device.

Each imaging session consisted of manually triggered trials with intertrial intervals of ≥2 min. Each trial consisted of 12 s of imaging in which an odor was presented in one 3 s pulse, using a custom-built multichannel auto-switching flow dilution olfactometer [54] with dedicated lines for each odor to avoid cross-contamination. Odorants were presented orthonasally to the animal at concentrations of either 0.1% or 1% s.v. Saturation was maintained by a flow (0.5 or 5 mL/min for 0.1% or 1% s.v., respectively) of filtered high-purity nitrogen (Airgas, NI ISP300, <0.1 ppm THC, H_2_O, and O_2_ contaminants) passing through passivated stainless steel spargers (IDEX health and science, A-243, 2 μm inlet filter) in PFA vials (Savillex 200-30-12) connected to the nose chamber (Fig 1A) via an air-dilution manifold. Odors were diluted using clean air (Airgas, AI UZ300, ultra-zero grade, <1 ppm THC, CO_2_, and CO contaminants) at a flow rate of 499.5 or 495 mL/min for 0.1% or 1% s.v., respectively, for a constant combined air-nitrogen-odor flow rate of 500 mL/min into the nose chamber. The nose chamber consisted of a 1 × 0.5 × 1” (WxHxD) Teflon block with two 5-mm ID channels 10 mm from the front of the block. This allowed connection of a 1/8” OD Teflon tube for diluted odor flow into one side and a vacuum connection (4 mm ID, 8 mm OD Tygon) for outflow on the opposing side. Flow of odorants was continuous and was removed via the vacuum (2.5 L/min) that was switched off for odorant delivery. A central channel of 6 mm ID connected the orthogonal odor-vacuum stream to the frontally placed nares. The tip of the mouse’s nose (including just the nares, OD 2 mm, Fig 1A) was placed just inside the chamber, whereby there was approximately 2 mm of space surrounding the entire nose for unrestricted flow. Odorants (Sigma-Aldrich) used were heptanone, hexanal, AA, carvone, MV, and heptanol (stored under nitrogen in the dark). Mice were freely breathing, which was continuously measured by a piezoelectric strip positioned on the animal's thorax.

Three *OMP-GCaMP6f* animals that had no prior exposure to odors were used for the mechanosensory air experiments. The clean (medical grade) air presented to the animal was not delivered via the multichannel auto-switching flow dilution olfactometer but was carbon filtered and delivered directly from a mass flow controller.

### Data analysis

Custom code written in LabView was used to extract the fluorescence traces from each trial. Frame subtraction was performed by selecting video frames just before and after odor presentation. This presented an image that highlighted regions that responded to odor stimulation. Multiple ROIs that resembled glomeruli were manually selected per mouse. This process was repeated for all trials, and additional ROIs were selected to accommodate all glomeruli that responded in a particular mouse for all odors and all concentrations. This generated an accumulated list of ROIs that could directly compare the responsiveness of all glomeruli across odors. The ROIs were used to extract mean fluorescence intensity traces from time series images of all trials. Graphpad Prism (version 7; GraphPad Software, CA) was use to generate plots and for statistical analysis. All data are presented as mean ± SEM.

### Data overview

For each trial, the optical imaging response traces (one .txt file for all ROIs per trial), ROI location (of the diagonally opposed corners of the rectangular area; no more than 40 per imaged OB area [lateral/dorsal] per animal; a .txt file), and sniffing and odor presentation timing traces (a .txt file) were analyzed in Matlab (R2018a, The Mathworks, MA) in batch mode. The total data set consisted of 6 animals with up to 48 trials each (6 odors × 2 conc × 3 repeats) and up to 80 ROIs (S1 Table).

All data were referenced to the very stable OB imaging sampling times (virtually jitter-free 30.00 fps), to which odor and sniffing data were resampled and then shifted for proper alignment, followed by truncation. Alignment was verified by comparing an optically imaged LED driven in parallel by the odor-on vacuum valve with that of the odor valve .txt file. The offset between the imaged data and odor/breathing data did not vary between trials.

### Spatiotemporal analysis of response patterns

The start of inhalation with the piezo sensor (once bandpass filtered) was a sharp downward deflection after a relatively shallow downward slope, verified by co-imaging of the thorax movement of a mouse. The timing thereof was determined by band-pass filtering (4th order, 1–10 Hz, zero phase shift) of the z-scored piezo voltage signal, followed by peak detection, from which the onset could reliably (1 sample jitter, 33.0 ms) be determined.

To determine the peak response amplitudes (% ΔF/F) and temporal parameters (including T90, see S1B Fig) of the glomerular responses, we used a custom algorithm that fitted the optical signals from each ROI to a double sigmoid function as described previously [44, 65]. The analysis allowed robust and objective measurement of response timing. Briefly, the signal from each ROI, after each identified inhalation, was band-pass filtered (second-order Butterworth, 0.1–7 Hz) followed by fourth-order Daubechies wavelet decomposition, soft thresholding of the coefficients at level 3, and then reconstruction. The onset time was defined based on the time of peak in the product of the first and the second derivatives of the optical signal. Starting at this time, each response was fitted (least-squares curve fitting) with a double-sigmoid function (a sigmoid rise followed by a sigmoid fall). The time of the peak of this response was defined as the peak in this fitted response function rather than the peak of the raw optical signal.

Next, we identified the inhalation onset time for each trial that evoked the first dorsal OB response during presentation of odor (“odor on response”; odor on from 3.4–6.4 s) by finding the largest response within the series of fitted responses averaged across ROIs for the period 2.8–4.5 s. We similarly determined the inhalation onset for largest mean lOB response (as we did not find any odor “off” responses in the dOB) per trial after the odor was turned off, between 6.0 and 9.0 s. These two inhalations were used to analyze the spatiotemporal responses for both the dOB and lOB (T10, T50, T90, Tpeak, and peak % ΔF/F) across glomeruli and the bulbs.

Global odor on response maps (Fig 2B) were established by correlating (Matlab function “corr”) for each mouse and odor the peak % ΔF/F of glomeruli with their location along each of their spatial dimensions (dOB: A-P and M-L [“laterality”: distance—in pixels—from midline]; lOB: A-P and D-V). Global maps of odor on response dynamics (Fig 3A–3C) were similarly made for T90 instead of response amplitude. The resulting correlation coefficients were subsequently averaged across mice. For pre-odor responses, the amplitudes and dynamics of each ROI were first averaged across all responses per trial, prior to making correlations (Fig 3B and 3C). Correlations of specific odor maps similarities (Fig 2C) were made by correlating odor on % ΔF/F responses from all identified ROIs across odors for dOB and lOB separately. z-Scores of each odor response were calculated for each trial by subtracting from an odor-evoked response the mean of the trial’s pre-odor responses and dividing this difference by the SD of the pre-odor responses:

$$z=\frac{{dFF}_{odor}-\mu ({dFF}_{pre-odor})}{\sigma ({dFF}_{pre-odor})}$$

Recruitment (Fig 4C) of glomeruli was quantified by first assessing whether each glomerular odor on response (peak % ΔF/F) was significantly larger than its own baseline breathing peak response amplitudes. Each odor on/off response was z-scored relative to its own response amplitudes prior to odor onset (see Fig 2D). A glomerulus responded (was “recruited”) when the likelihood was <1% (i.e., *P* < 0.01).

The odor off histograms (Fig 5D) were based on the off response amplitude (peak % ΔF/F) of each glomerulus. This was divided by the off response amplitude (peak % ΔF/F) during breathing trials with no air or vacuum. As no stimulus was delivered for the breathing trial, the off response was the taken as the peak response at the same timepoint at in the stimulus trials. The histograms are based on all stimulus-delivered trials combined.

We attempted to record intranasal pressure changes via a nasal cannula, but we were unable to reliably detect any changes unless the nose was in much closer proximity to the odor delivery tube than during the imaging experiments. Hence, to reliably measure the relative pressure change for the vacuum on and vacuum off responses (S4A Fig), a tube (31 mm long, 6.0 mm OD, 3.0 mm ID) was connected to the positive sensing orifice of a Buxco (TRD 5700) pressure sensor. The opposite edge of the tube was placed just inside the odor delivery tube (ID 7.1 mm) used during the in vivo study, thereby leaving a wide gap, like the in vivo setup. Buxco sensor output was band-pass filtered (0–1 kHz), amplified (1000×), and the resulting voltage was recorded for steady-state air and vacuum flow rate combinations (S4A Fig, and calculated changes in B). Dynamic conditions settled to steady-state values nearly instantaneously. To explore pressure change-OB response functions, the mean of the z-scored response across all glomeruli (dOB: 99; lOB: 47; *n* = 3 mice) was calculated. Out of a total of 1,584 dOB responses (16 flow conditions × 99 glomeruli) and 752 lOB responses (16 × 47), we removed 4 outliers (z-score of z-score was < −4 or > 4, based on distribution of all z-scores for vacuum on and off separately and dOB and lOB separately) from each dataset (S4C and S4D Fig). For plotting of the functions (Fig 6), the responses were uniformly offset by the mean of the respective z-scores for no flow change condition (“0–0 [room]”; z = 0.9 for dOB and z = 1.5 for lOB), which had a positive bias due to our sniff-response–selecting algorithm picking the largest response averaged across glomeruli within a defined response period.

## Supporting information

S1 Fig Glomerular dynamics across dOB and lOB. (A) Amplitude responses of glomeruli for heptanone (left) and hexanal (right) and their position across the A-P dimension in the dOB, demonstrating their linear correlations, as shown in Fig 2B. B Generic fluorescence trace of a glomerulus displaying the determination of the T90 of a response. (C) The average T90 responses over all glomeruli for each odor in the dorsal bulb (AA, heptanol, and hexanal 5 animals; all others 6 animals) and lOB (AA, heptanol, and hexanal 5 animals; all others 6 animals). (D) Comparison of the T90 responses of glomeruli in the anterior and posterior dOB. (E) Comparison of the T90 responses of glomeruli in the dorsal and ventral lOB. Statistics represent two-way ANOVA (odor × OB region) with Bonferroni’s multiple comparisons test. ♦ denotes statistical significance between dorsal and lateral T90 for all odors. Error bars are SEM. ♦ *P* < 0.05, ♦♦ *P* < 0.01, ♦♦♦ *P* < 0.001, ♦♦♦♦ *P* < 0.0001. Underlying data for this figure can be found in S1 Data.
(TIFF)

S2 Fig Glomerular peak responses across dOB and lOB. (A) Color-scaled ΔF/F responses for ROIs for all odors. ΔF/F are scaled between 0 and 43% (1 animal). (B) Color-scaled ΔF/F responses for ROIs for all odors. ΔF/F are scaled to each odors maximum value (1 animal). Underlying data for this figure can be found in S1 Data.
(TIFF)

S3 Fig Glomerular dynamics across dOB and lOB. (A) Color-scaled T90 responses for ROIs for all odors. T90 are scaled between 0 and 350 ms (1 animal). (B) Color-scaled T90 responses for ROIs for all odors. T90 are scaled to each odors maximum value (1 animal). Underlying data for this figure can be found in S1 Data.
(TIFF)

S4 Fig Glomerular responses in dOB and lOB versus air flow conditions. (A) The differential pressure change of the individual (positive) clean air and (negative) vacuum flow rates and the differential pressure change of them combined (see Methods). Arrows demonstrate a relatively large reduction of the pressure drop of both clean air and vacuum compared to vacuum alone. (B) A comparison of net flow rate and differential pressure at all air and vacuum flow rates. (C) The z-score values of all glomeruli in response to either vacuum on or vacuum off at all flow rates in the dOB. (D) Same as C but in the lOB, 3 animals, dOB: 99 glomeruli, lOB: 47 glomeruli. Underlying data for this figure can be found in S1 Data.
(TIFF)

S5 Fig The dOB lacks the pressure sensitivity that the lOB displays. The z-scores of the dOB glomerular responses during only clean air flow (red) and also vacuum (grey), organized from anterior to posterior, for different clean air flow rates (0.5, 0.25, 0.1, 0.05, and 0.005 L/min) and vacuum rates (2.5, 1.25, and 0 L/min) and room air (3 animals, 47 glomeruli). Linear correlation fits are indicated, 3 animals, 99 glomeruli. Underlying data for this figure can be found in S1 Data.
(TIFF)

S1 Table Animal number, glomerular number, and trial number for both the dOB and lOB for 0.1, 1% odor concentration and air. (TIFF)

S2 Table Global correlation values of odor on responses across spatial dimensions. (A) In the dOB, A-P, and M-L dimensions. (B) In the lOB, A-P and D-L dimensions.
(TIFF)

S3 Table Pearson correlation values of glomerular responses for all odors. (A) In the dOB. (B) In the lOB.
(TIFF)

S4 Table Average of T90 responses for all glomeruli in the dOB and lOB. (TIFF)

S5 Table Linear correlations of T90 with location along each spatial dimension prior to odor presentation (pre-odor breathing responses) and upon odor presentation (post odor). (A) In the dorsal bulb. (B) In the lateral bulb.
(TIFF)

S6 Table Correlation between the glomerular response amplitudes and the T90 responses of these glomeruli in the dOB and the lOB. (TIFF)

S7 Table The percentage of glomeruli significantly activated by 0.1% odors normalized relative to 1% in the dOB and lOB. (TIFF)

S8 Table The average amplitudes of all glomeruli responding to 0.1 and 1% for all odors. (A) In the dOB. (B) In the lOB.
(TIFF)

S9 Table The slope and significance values for the linear regressions of the z-scores for glomerular responses and their location along the D-V dimension of the lOB in response to air and vacuum. (TIFF)

S1 Data (XLSX)

## Abbreviations

2-DG: 2-deoxyglucose
A-P: anterior-posterior
AA: amyl acetate
D-L: dorso-lateral
D-V: dorso-ventral
dOB: dorsal olfactory bulb
fMRI: functional magnetic resonance imaging
HEPA: high-efficiency particulate air
lOB: lateral olfactory bulb
M-L: medio-lateral
MV: methyl valerate
OB: olfactory bulb
OE: olfactory epithelium
OR: olfactory receptor
OSN: olfactory sensory neuron
PACUC: Pierce Animal Care and Use Committee
ROI: region of interest
s.v.: saturated vapor
TAAR: trace amine-associated receptor
