# Supplementary figures and images for: Spatiotemporal dynamics of odor responses in the lateral and dorsal olfactory bulb
Source: PLoS Biol. 2019 Sep 18;17(9):e3000409. doi: 10.1371/journal.pbio.3000409 (PMC6768483; doi:10.1371/journal.pbio.3000409)

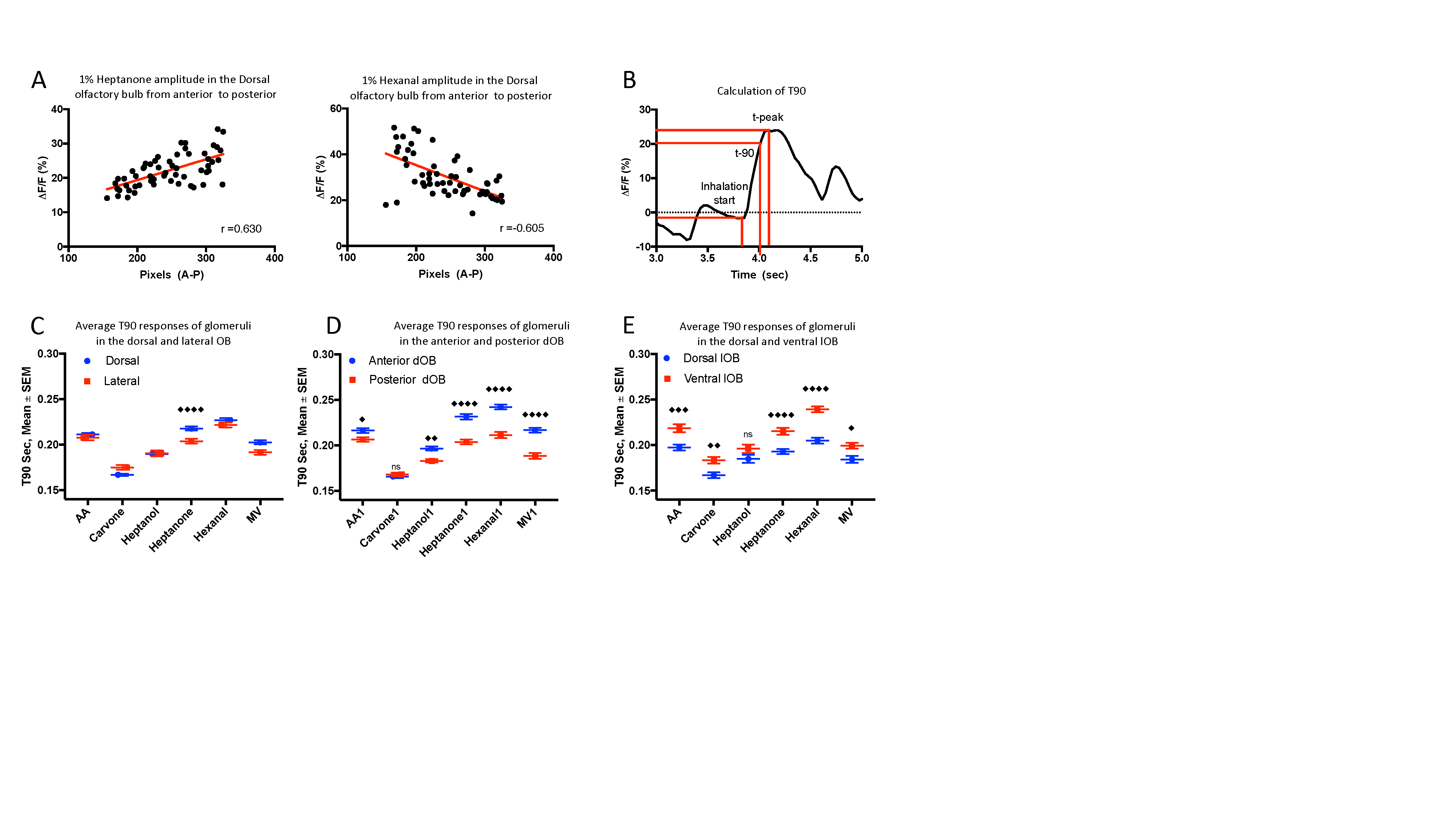

Supplement: S1 Fig — (A) Amplitude responses of glomeruli for heptanone (left) and hexanal (right) and their position across the A-P dimension in the dOB, demonstrating their linear correlations, as shown in Fig 2B. B Generic fluorescence trace of a glomerulus displaying the determination of the T90 of a response. (C) The average T90 responses over all glomeruli for each odor in the dorsal bulb (AA, heptanol, and hexanal 5 animals; all others 6 animals) and lOB (AA, heptanol, and hexanal 5 animals; all others 6 animals). (D) Comparison of the T90 responses of glomeruli in the anterior and posterior dOB. (E) Comparison of the T90 responses of glomeruli in the dorsal and ventral lOB. Statistics represent two-way ANOVA (odor × OB region) with Bonferroni’s multiple comparisons test. ♦ denotes statistical significance between dorsal and lateral T90 for all odors. Error bars are SEM. ♦ P < 0.05, ♦♦ P < 0.01, ♦♦♦ P < 0.001, ♦♦♦♦ P < 0.0001. Underlying data for this figure can be found in S1 Data. (TIFF) [file pbio.3000409.s001.tiff]

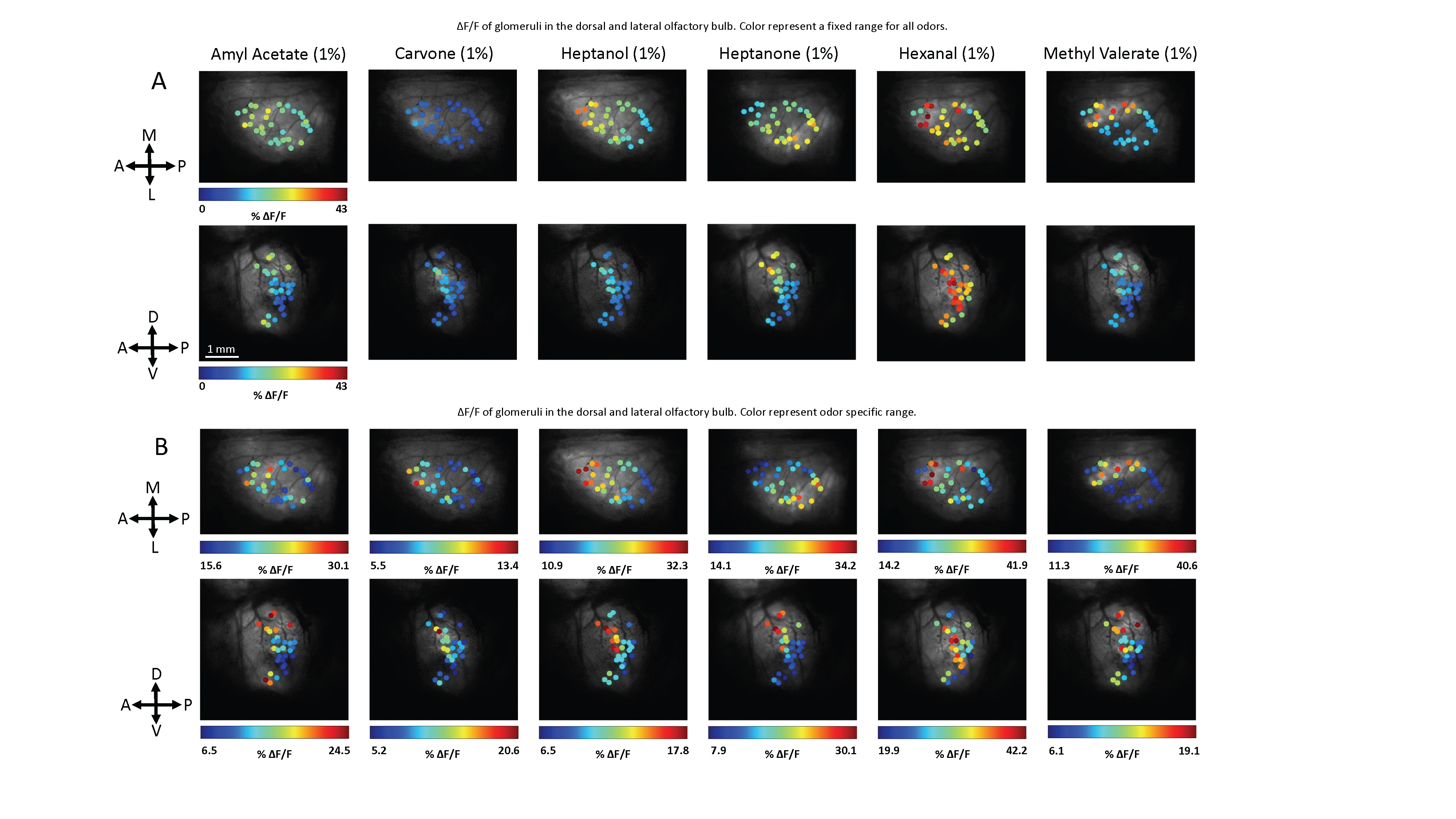

Supplement: S2 Fig — (A) Color-scaled ΔF/F responses for ROIs for all odors. ΔF/F are scaled between 0 and 43% (1 animal). (B) Color-scaled ΔF/F responses for ROIs for all odors. ΔF/F are scaled to each odors maximum value (1 animal). Underlying data for this figure can be found in S1 Data. (TIFF) [file pbio.3000409.s002.tiff]

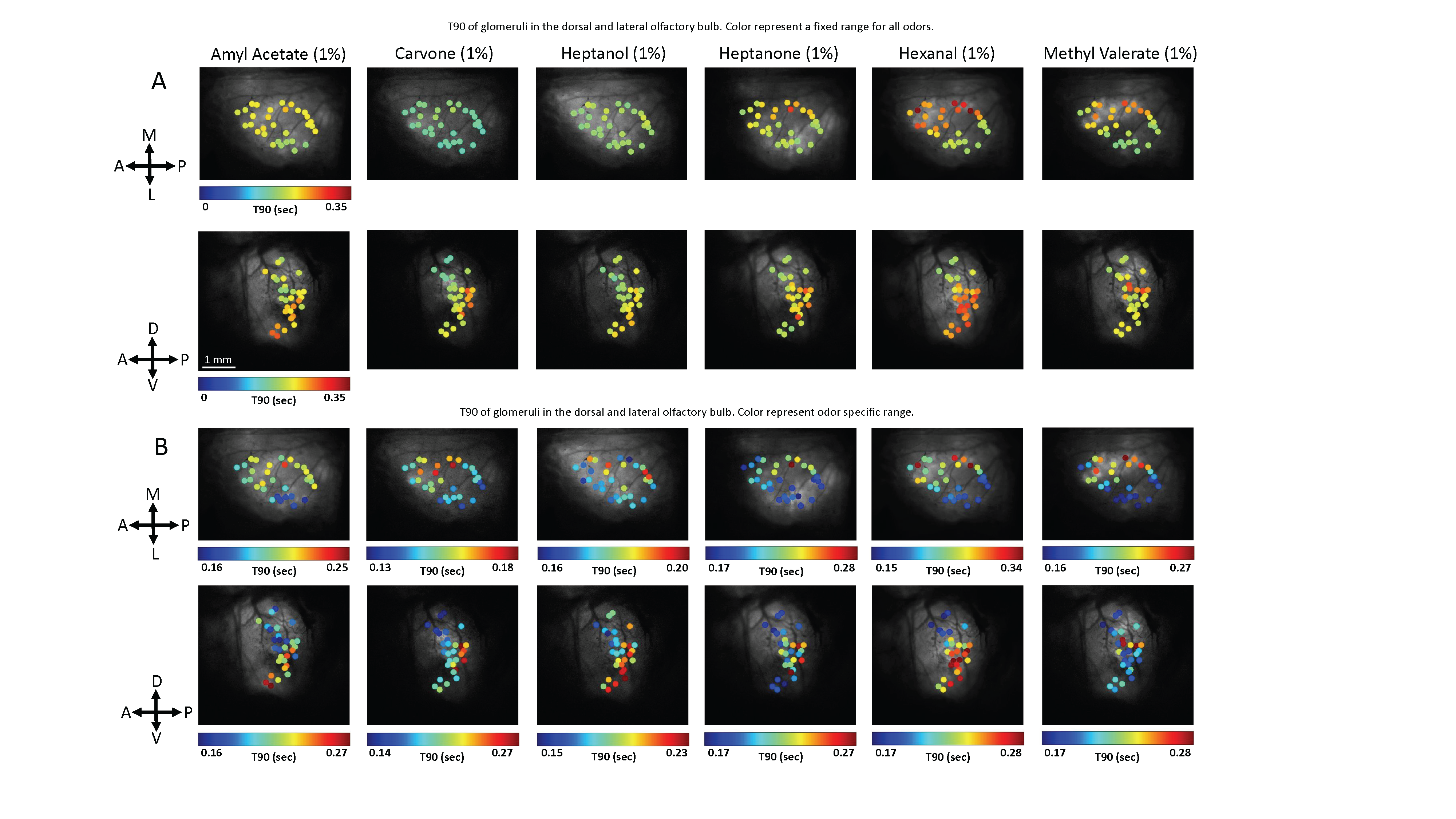

Supplement: S3 Fig — (A) Color-scaled T90 responses for ROIs for all odors. T90 are scaled between 0 and 350 ms (1 animal). (B) Color-scaled T90 responses for ROIs for all odors. T90 are scaled to each odors maximum value (1 animal). Underlying data for this figure can be found in S1 Data. (TIFF) [file pbio.3000409.s003.tiff]

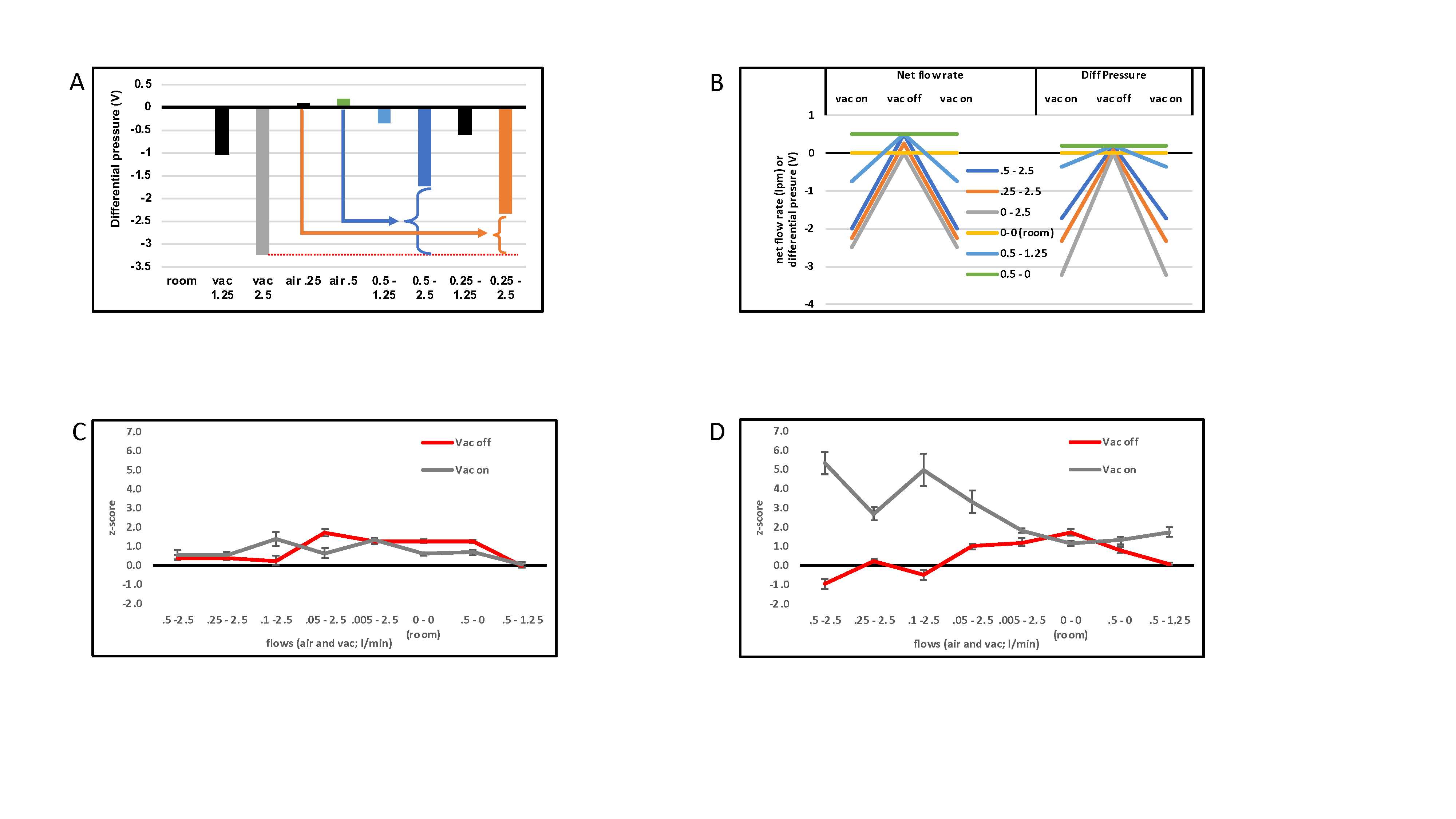

Supplement: S4 Fig — (A) The differential pressure change of the individual (positive) clean air and (negative) vacuum flow rates and the differential pressure change of them combined (see Methods). Arrows demonstrate a relatively large reduction of the pressure drop of both clean air and vacuum compared to vacuum alone. (B) A comparison of net flow rate and differential pressure at all air and vacuum flow rates. (C) The z-score values of all glomeruli in response to either vacuum on or vacuum off at all flow rates in the dOB. (D) Same as C but in the lOB, 3 animals, dOB: 99 glomeruli, lOB: 47 glomeruli. Underlying data for this figure can be found in S1 Data. (TIFF) [file pbio.3000409.s004.tiff]

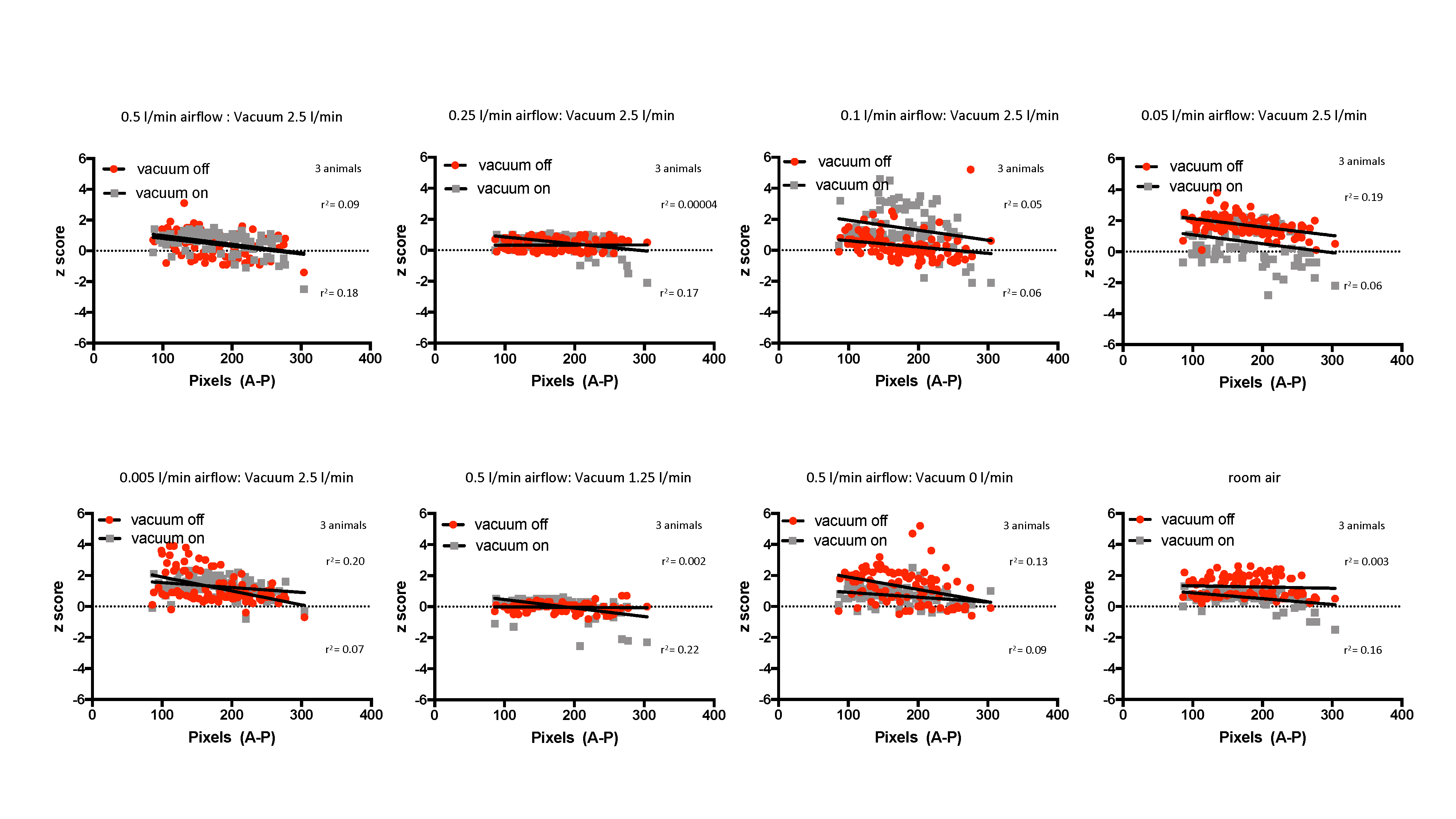

Supplement: S5 Fig — The z-scores of the dOB glomerular responses during only clean air flow (red) and also vacuum (grey), organized from anterior to posterior, for different clean air flow rates (0.5, 0.25, 0.1, 0.05, and 0.005 L/min) and vacuum rates (2.5, 1.25, and 0 L/min) and room air (3 animals, 47 glomeruli). Linear correlation fits are indicated, 3 animals, 99 glomeruli. Underlying data for this figure can be found in S1 Data. (TIFF) [file pbio.3000409.s005.tiff]

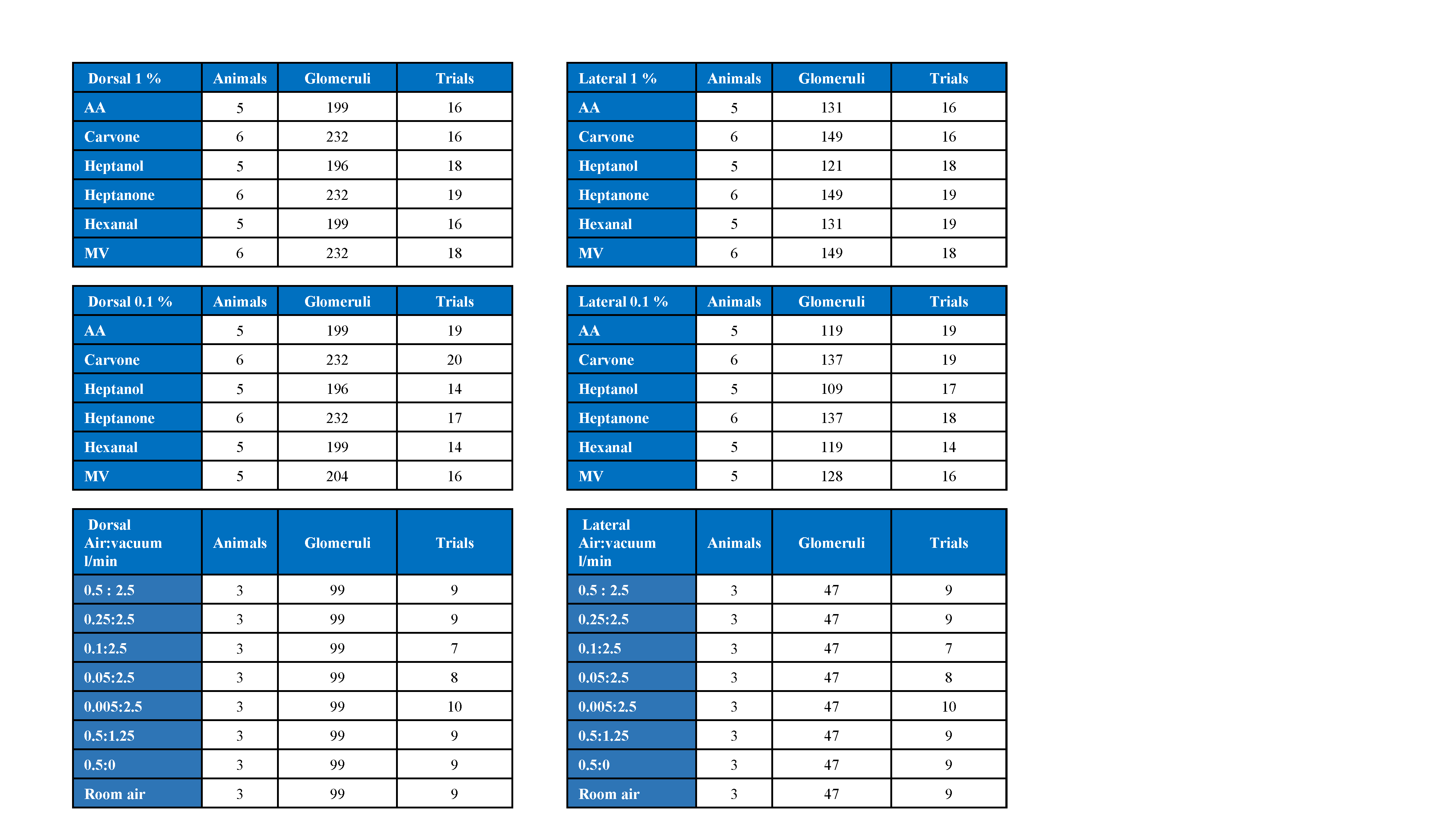

Supplement: S1 Table — (TIFF) [file pbio.3000409.s006.tiff]

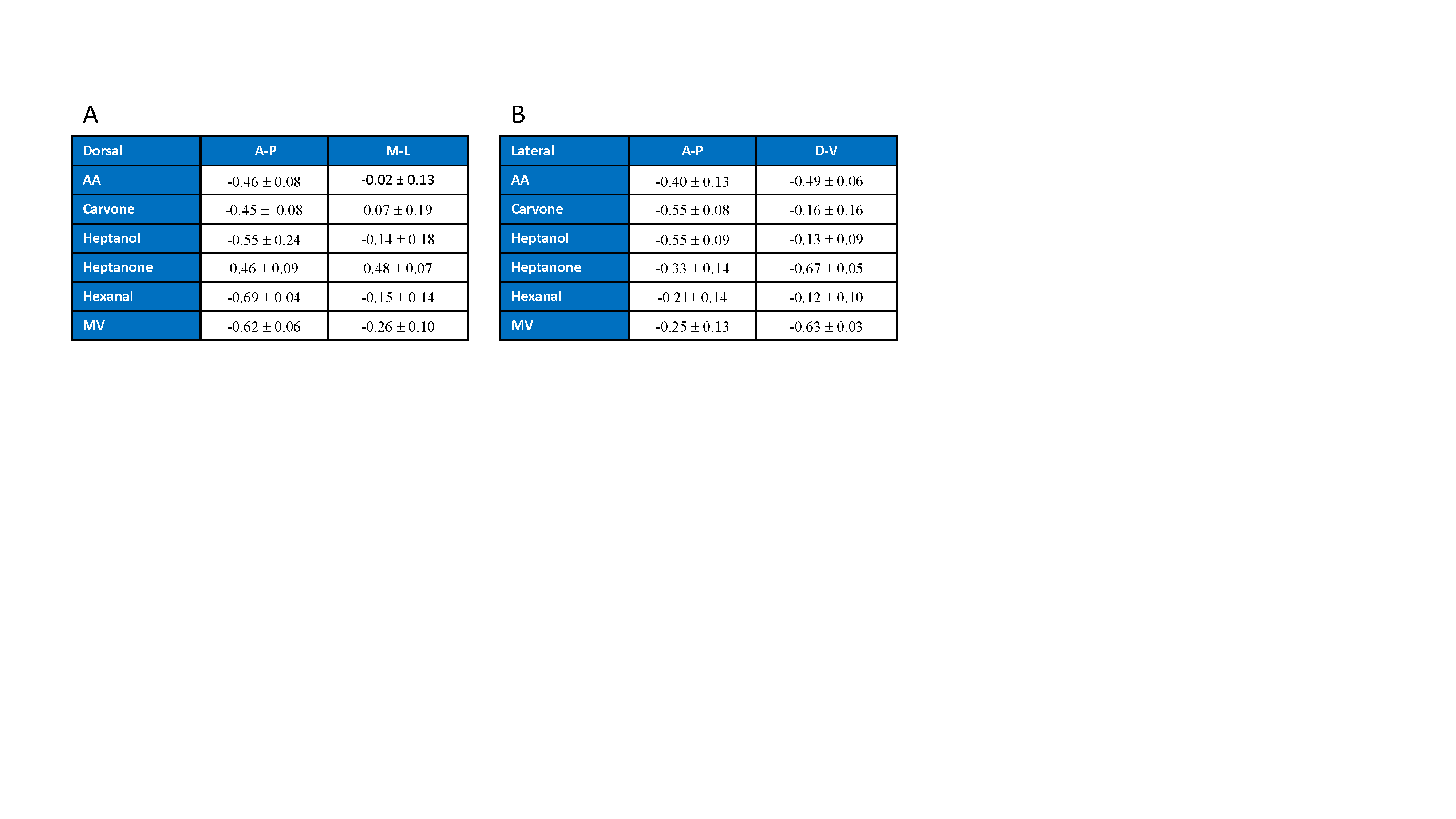

Supplement: S2 Table — (A) In the dOB, A-P, and M-L dimensions. (B) In the lOB, A-P and D-L dimensions. (TIFF) [file pbio.3000409.s007.tiff]

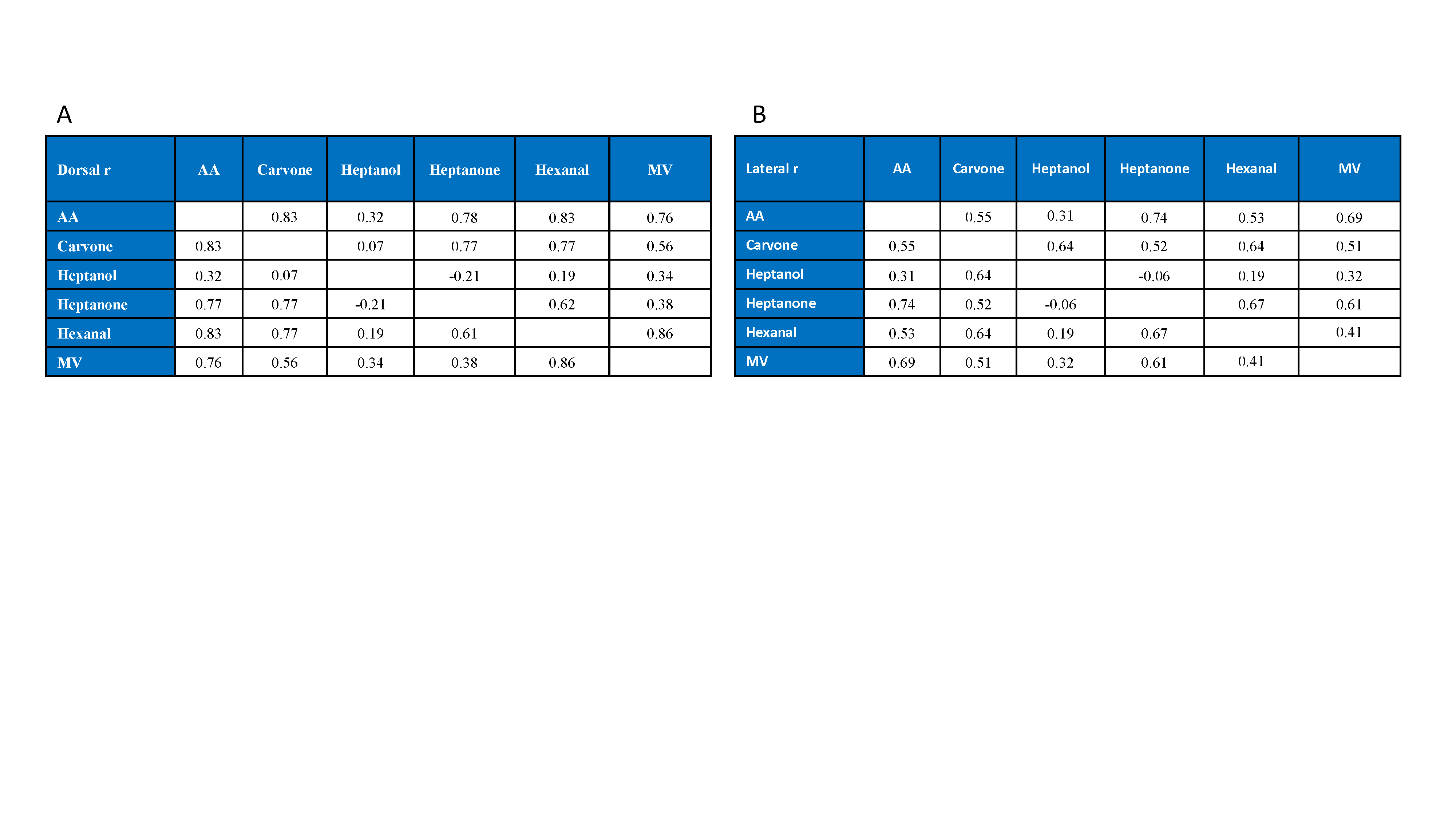

Supplement: S3 Table — (A) In the dOB. (B) In the lOB. (TIFF) [file pbio.3000409.s008.tiff]

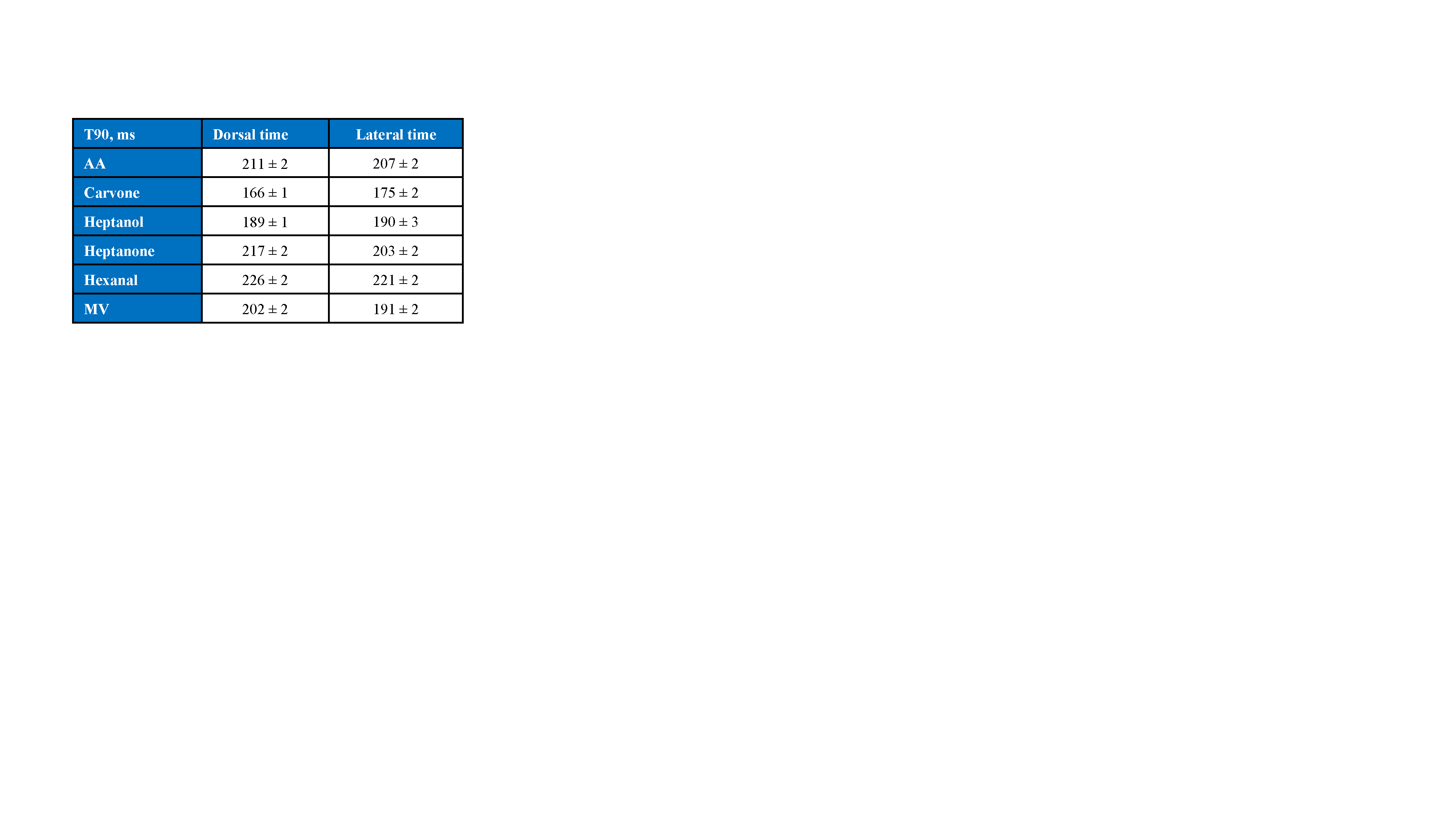

Supplement: S4 Table — (TIFF) [file pbio.3000409.s009.tiff]

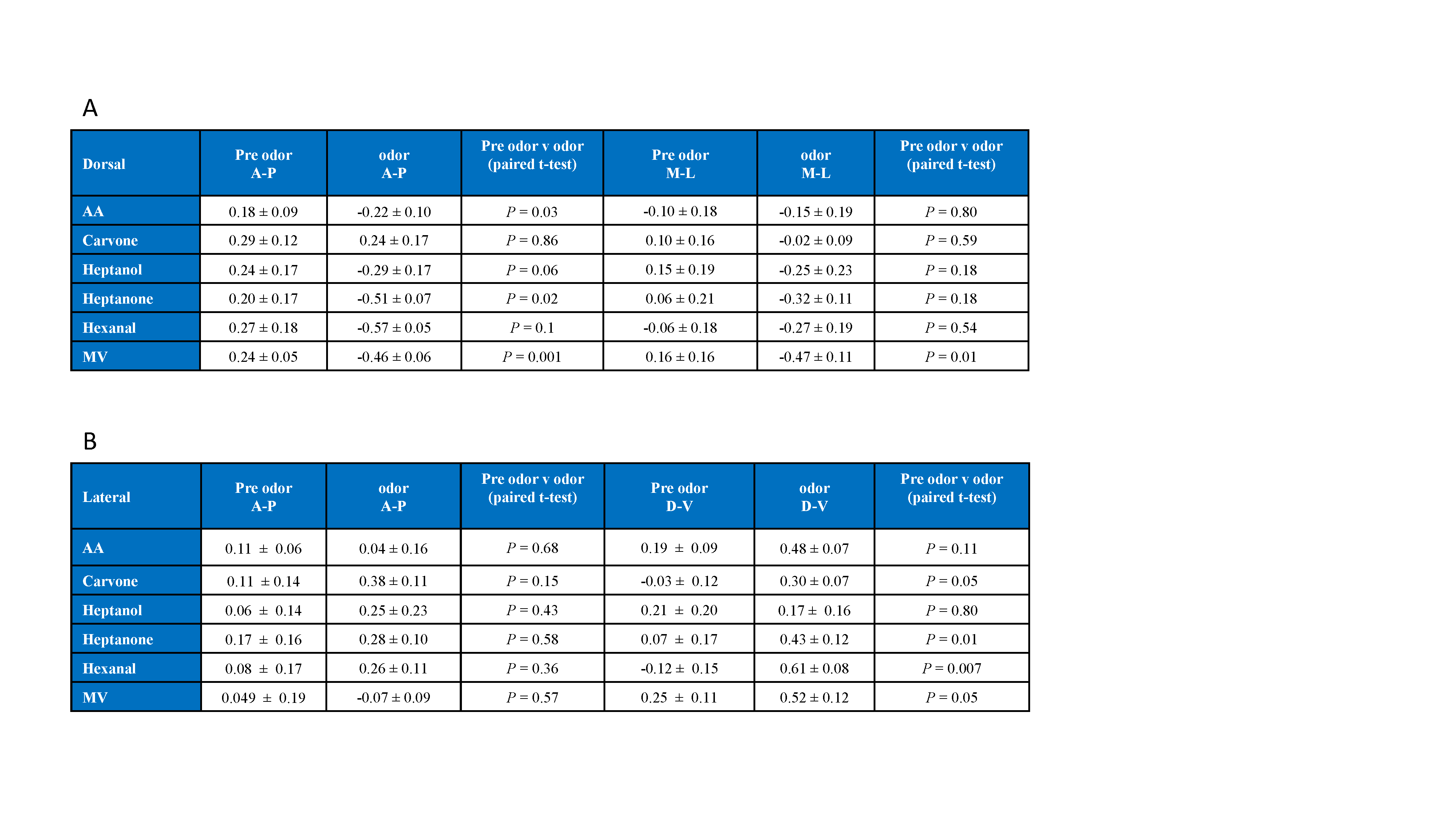

Supplement: S5 Table — (A) In the dorsal bulb. (B) In the lateral bulb. (TIFF) [file pbio.3000409.s010.tiff]

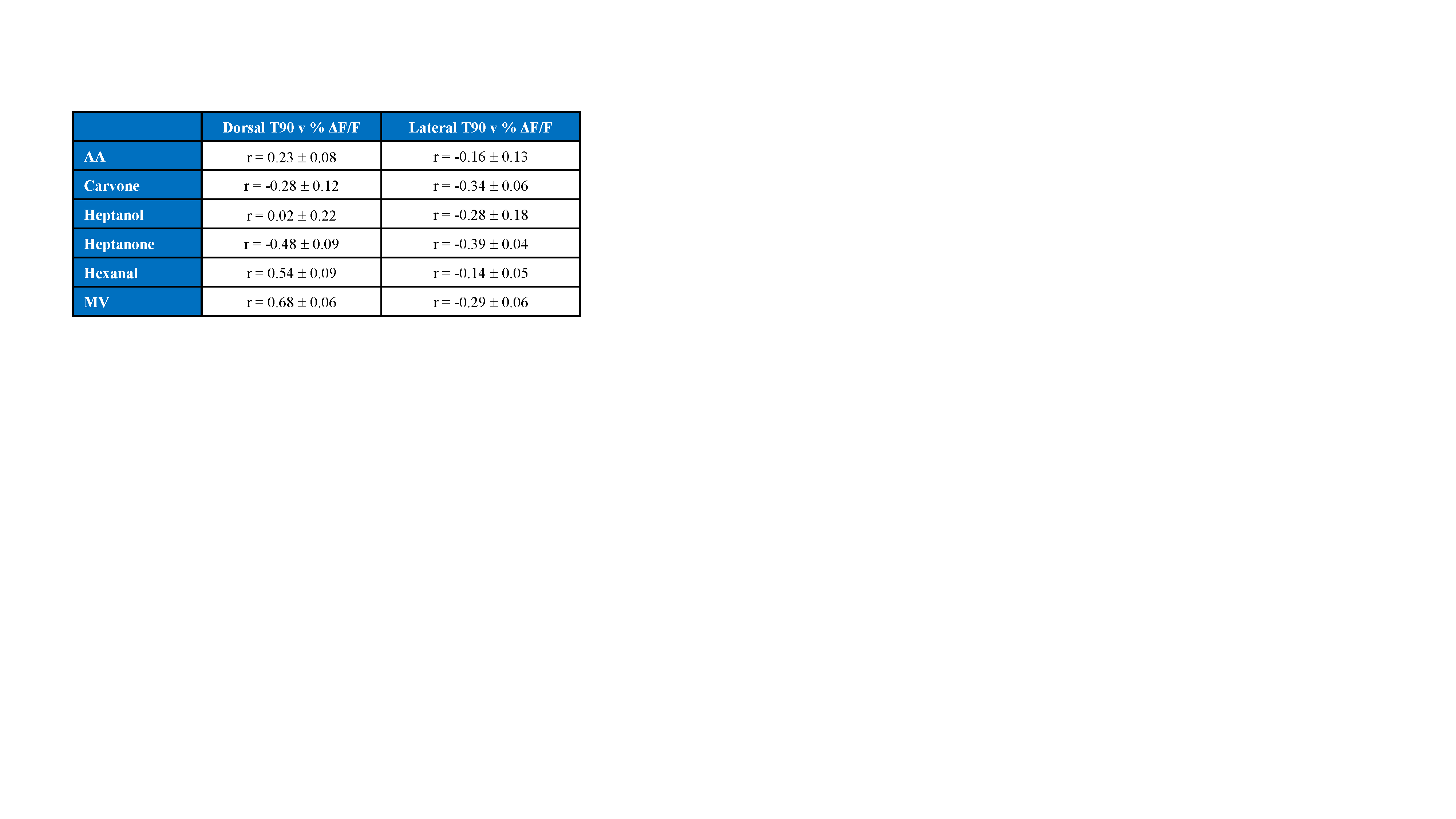

Supplement: S6 Table — (TIFF) [file pbio.3000409.s011.tiff]

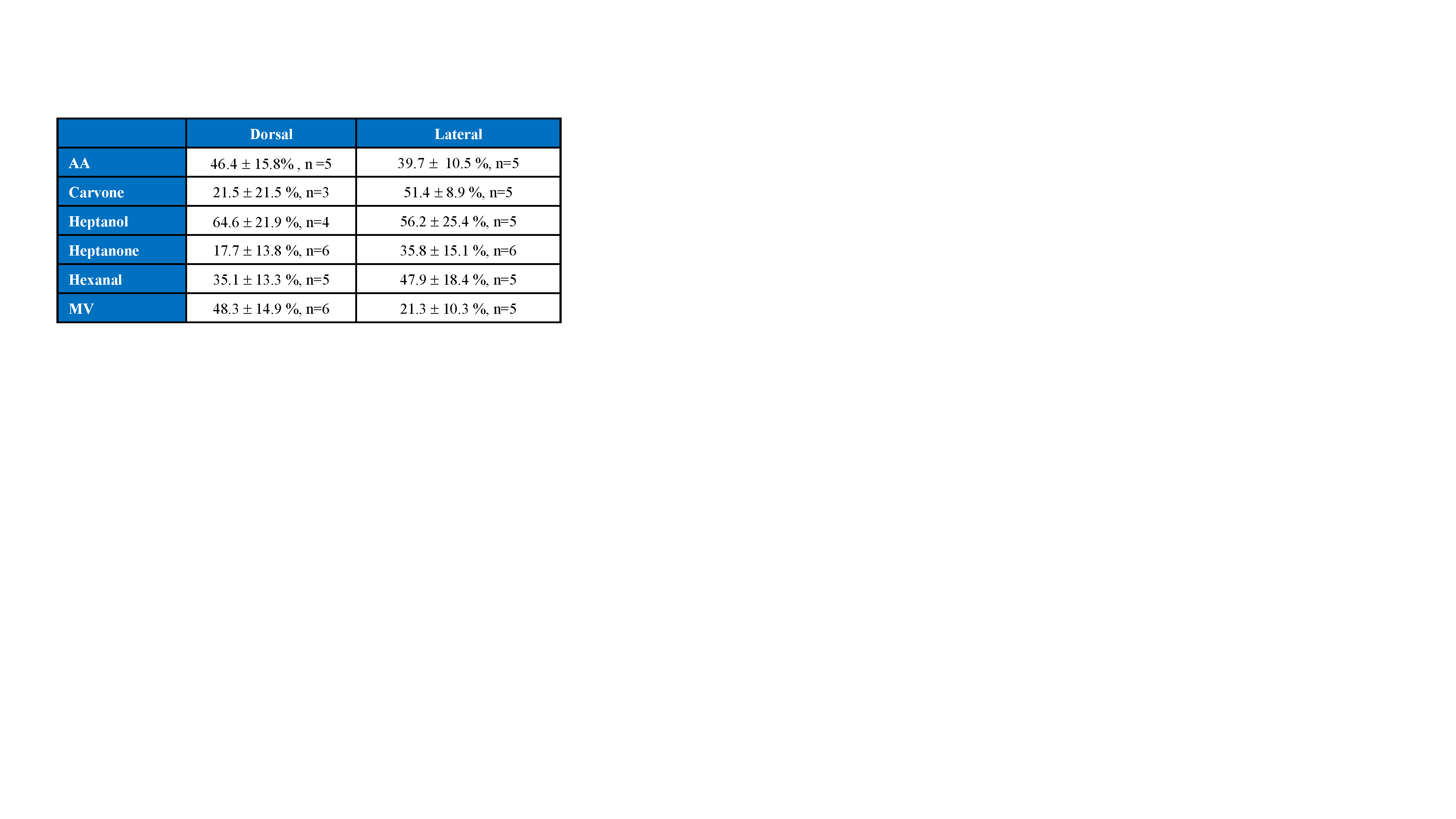

Supplement: S7 Table — (TIFF) [file pbio.3000409.s012.tiff]

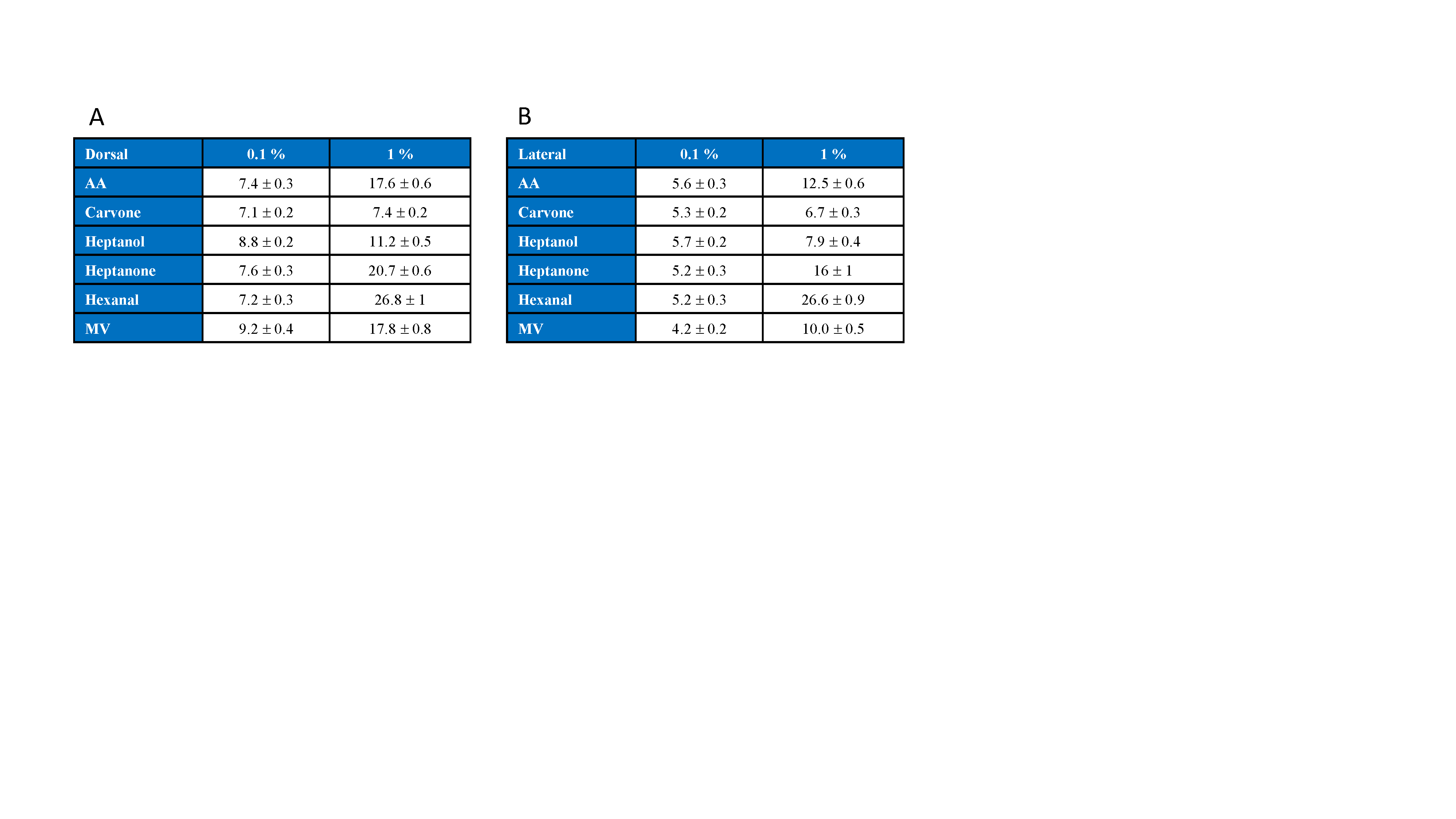

Supplement: S8 Table — (A) In the dOB. (B) In the lOB. (TIFF) [file pbio.3000409.s013.tiff]

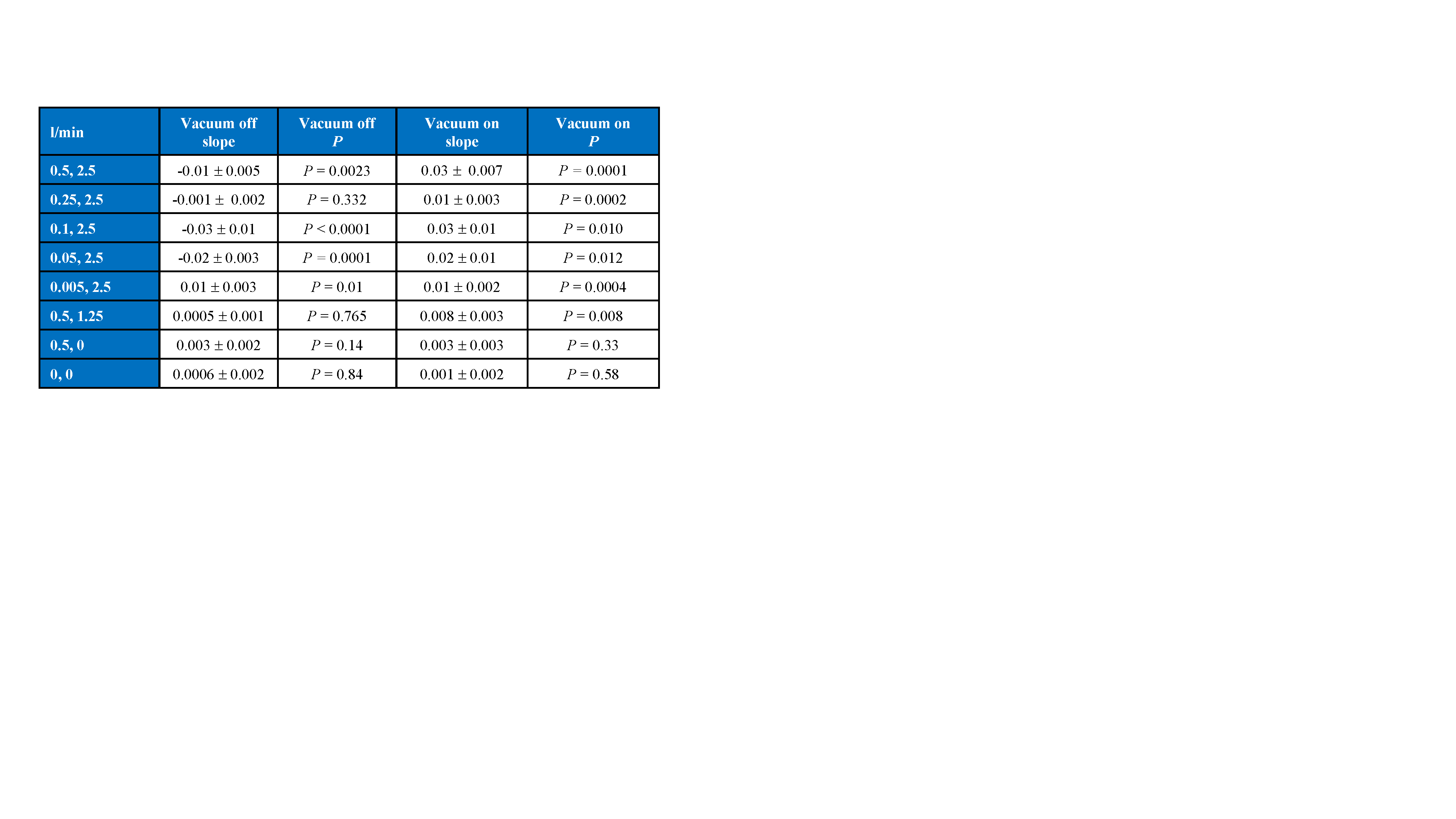

Supplement: S9 Table — (TIFF) [file pbio.3000409.s014.tiff]
